# Exome sequencing improves the molecular diagnostics of paediatric unexplained neurodevelopmental disorders

**DOI:** 10.1186/s13023-024-03056-6

**Published:** 2024-02-06

**Authors:** Marketa Wayhelova, Vladimira Vallova, Petr Broz, Aneta Mikulasova, Jan Smetana, Hana Dynkova Filkova, Dominika Machackova, Kristina Handzusova, Renata Gaillyova, Petr Kuglik

**Affiliations:** 1 Department of Experimental Biology, Faculty of Science, Masaryk University, Brno, Czech Republic; 2 Centre of Molecular Biology and Genetics, University Hospital Brno, Brno, Czech Republic; 3 Department of Biology and Medical Genetics, 2nd Faculty of Medicine, Charles University Prague and University Hospital Motol, Prague, Czech Republic; 4 Biosciences Institute, Faculty of Medical Sciences, Newcastle University, Newcastle Upon Tyne, UK; 5 Department of Medical Genetics and Genomics, University Hospital Brno, Brno, Czech Republic

**Keywords:** Exome sequencing, Neurodevelopmental disorders, Sequence variant, Copy-number variation

## Abstract

**Background:**

Neurodevelopmental disorders (NDDs) and/or associated multiple congenital abnormalities (MCAs) represent a genetically heterogeneous group of conditions with an adverse prognosis for the quality of intellectual and social abilities and common daily functioning. The rapid development of exome sequencing (ES) techniques, together with trio-based analysis, nowadays leads to up to 50% diagnostic yield. Therefore, it is considered as the state-of-the-art approach in these diagnoses.

**Results:**

In our study, we present the results of ES in a cohort of 85 families with 90 children with severe NDDs and MCAs. The interconnection of the *in-house* bioinformatic pipeline and a unique algorithm for variant prioritization resulted in a diagnostic yield of up to 48.9% (44/90), including rare and novel causative variants (41/90) and intragenic copy-number variations (CNVs) (3/90). Of the total number of 47 causative variants, 53.2% (25/47) were novel, highlighting the clinical benefit of ES for unexplained NDDs. Moreover, trio-based ES was verified as a reliable tool for the detection of rare CNVs, ranging from intragenic exon deletions (*GRIN2A, ZC4H2* genes) to a 6-Mb duplication. The functional analysis using PANTHER Gene Ontology confirmed the involvement of genes with causative variants in a wide spectrum of developmental processes and molecular pathways, which form essential structural and functional components of the central nervous system.

**Conclusion:**

Taken together, we present one of the first ES studies of this scale from the central European region. Based on the high diagnostic yield for paediatric NDDs in this study, 48.9%, we confirm trio-based ES as an effective and reliable first-tier diagnostic test in the genetic evaluation of children with NDDs.

**Supplementary Information:**

The online version contains supplementary material available at 10.1186/s13023-024-03056-6.

## Introduction

Neurodevelopmental disorders are a genetically heterogeneous group of conditions affecting the normal development of the central nervous system (CNS), with an adverse prognosis for the quality of intellectual and social abilities, as well as daily functioning. With a reported prevalence of 1–2% of live births, they represent one of the most discussed current health and social issues [1]. Their high heterogeneity is reflected in the genetic and phenotypic overlap of distinct disorders, making them difficult to differentiate clinically. The symptoms typically begin in childhood and persistently affect development. Intellectual disability of varying degrees, with isolated occurrence or accompanied by multiple congenital abnormalities affecting intellectual and somatic development, is widely reported as the most prominent clinical feature.

The current guidelines for the genetic evaluation of individuals with NDDs and MCAs still recommend the chromosomal microarray analysis (CMA) as the first-tier molecular diagnostic test which overcomes the traditional karyotyping using G-banding [2, 3]. However, other diagnostic test as fragile X testing or metabolic tests may be conclusive in those cases with suggestive and prominent clinical symptoms [4].

According to the current information obtained from sysID database, there are more than 1500 known and more than 1200 candidate genes of which rare variations can be responsible for the phenotypic manifestation of abnormal brain development and functioning [5]. AutDB as an “autism information portal” summarizes the information about more than 1200 genes involved in the phenotypes of autism spectrum disorders (ASD) [6]. The significant genetic overlap exists among neurodevelopmental and neuropsychiatric disorders due to shared signalling and developmental pathways [7].

Trio-based ES involving affected individuals and their parents was recently proposed as the most effective molecular diagnostic approach for families with clinical features of Mendelian disorders including NDDs [8, 9]. Nowadays it is a rapidly evolving method for the simultaneous detection of sequence variants and copy-number variations (CNVs) [10]. Therefore, the individuals with a family history of disease or with its solitary occurrence can avoid a low-yield or time-consuming diagnostic tests by undergoing this effective and powerful analysis. The rapid and accurate molecular diagnosis improves the short- and long-term disease management with reduced complications. The conclusive outputs of ES can specify the prognosis of disease and improve the quality of life regarding to the optimised and targeted, even symptomatic therapy. Moreover, the elucidation of the molecular basis of the abnormal phenotype can facilitate family-focused genetic counselling with reproductive outcomes [11, 12]. Therefore, it is not surprising that high-throughput genomic analyses as ES and GS are becoming preferable molecular diagnostic approaches in the genetic evaluation of individuals with NDDs and MCAs throughout the clinical laboratories and medical centres worldwide [10, 11].

However, the option of ES for the molecular diagnostics of unexplained NDDs and MCAs is mostly funded by research studies and grants so far in this country, therefore there are not any general recommendations or guidelines for ES as a standard genetic test. Instead, the phenotype-driven (virtual) gene panel “next generation” sequencing (NGS) encompassing the limited number of genes for specific entities, are widely offered, and covered by public health insurance based on the referral from the clinical geneticists. Their implementation to the molecular diagnostics of rare diseases reduces the turnaround time together with the maintenance of the comparable diagnostic yield as clinical ES [13].

This study presents the results of trio-based ES in the group of 90 children with NDDs from 85 families, reaching a diagnostic yield of 48.9% (44/90) for pathogenic single-nucleotide variants, short insertions/deletions, and intragenic CNVs.

## Results

### Quality control parameters of ES

Family-based ES was performed in 90 paediatric patients with NDDs and MCAs, their parents and/or in their unaffected siblings. Before variant prioritization and analysis, the QC metrics of processed sequencing outputs were calculated and inspected (Additional file 1). Briefly, on average, more than 81 million unique reads per sample were mapped to the reference genome GRCh38/hg38 primary assembly. Approximately 98% of targeted bases were covered to at least 30X and median target coverage was calculated as 97X. The average proportion of flagged PCR duplicates was only 14% and the average uniformity reached 1.41 which is a good assumption for CNV analysis. Moreover, no considerable differences in QC metrics between index and pooled samples have been observed (Additional file 1). The fraction of all target bases achieving 30X or greater coverage was calculated as 96% of all target bases in index cases (n = 18) and pooled samples (n = 14) as well.

### *Diagnostic yield and *in silico* functional characterization*

The effective process of the variant prioritization and interpretation assessed the molecular diagnosis in 48.9% cases (44/90) of paediatric NDDs and associated MCAs. The causative SNVs and indels were identified in 45.6% (41/90) of cases whereas the intragenic CNVs were found in much lower proportion, 3.3% (3/90).

The Gene Ontology (GO) analysis using the PANTHER™ Classification system with GO annotation was performed for the characterization of the gene set with causative variants. The set of 41 genes was categorized in five PANTHER™ Ontologies: Molecular Function (output for 10 categories), Biological Process (10 categories), Cellular Component (3 categories), Protein Class (12 categories), Pathway (45 categories) after manual curation. Almost half of genes (20/41) with causative variants are involved in binding and 34.1% (14/41) perform a catalytic activity on a molecular level. The disruption of cellular functioning is predicted due to the causative variants in 65.9% (27/41) of genes and 46.3% (19/41) of genes play a crucial role in metabolic processes. However, 63.4% (26/41) of genes remained uncategorized after the analysis of 177 curated, mostly signalling, pathways in the ontology PANTHER™ Pathway, indicating their broad structural and functional diversity as well as still uncharacterized involvement in the cell structure and functioning. Vice versa, 19.5% (8/41) genes were categorized in at least two pathways (Additional files 2; 3, Sheet 1–5). The PANTHER™ statistical overrepresentation test in these five PANTHER™ Ontologies assessed the overrepresentation of the analysed 41 genes: Biological Process (overrepresentation in 60 categories), Molecular Function (33 categories), Cellular Component (8 categories), Protein Class (one category), Pathway (6 categories including category “unclassified”) and Reactome Pathways (2 categories) after manual curation (Additional file 4, Sheet 1–5).

### Pathogenic sequence variants

More than half of causative variants (22/43) were of de novo origin (de novo variants observed in two pairs of monozygotic twins were counted once per one family). Approximately 40% (17/43) were of a familial origin whereas five variants were of paternal origin, including two of them confirmed as mosaic of ~ 11% (*CTNNB1* gene) and ~ 15% (*DYNC1H1* gene), respectively, in paternal DNA samples. The comparable degrees of mosaicism were found in the paternal samples of buccal swabs, ~ 13% for the *CTNNB1* gene variant and ~ 10% for the *DYNC1H1* gene variant, respectively. Other two variants (*GRIN2A* and *NFIB* genes) were inherited from affected fathers and one variant (*CACNA1A* gene) was inherited from apparently unaffected fathers. Two variants (*GCH1* and *KCNC3* genes*)* and four hemizygous X-linked variants (*EDA, OPHN1*, *SLC16A2* and *PTCHD1* genes) were inherited from asymptomatic mothers. The detailed analysis of genotype–phenotype correlation suggested four variants of maternal origin (*GABRB2, PBX1, CUX2* and *CACNA1C* genes) as the cause of abnormal phenotypes not only in probands but also in their mothers. Other two cases (2/43) had causative variants in the compound heterozygosity (*BLM* and *NACLN* genes), resulting in the clinical manifestation of associated rare, autosomal recessive (AR) disorders. Of two de novo mitochondrial causative variants, one was identified as homoplasmic (~ 95%, *MT-ATP6* gene) in the affected individual. Surprisingly, another LP variant (*MT-CO3* gene) was found in ~ 20% heteroplasmy in affected monozygotic twins. The origin of causative variants was not resolved in one case with the occurrence of two causative variants (*TCOF1* and *CPA6* genes), since the paternal DNA sample was not available. Two recurrently mutated genes in unrelated index cases were enriched in the list of causative variants, *SHANK3* (3 cases) and *RAI1* (2 cases). The causative and candidate variants and their pathogenicity are summarized (Additional file 5).

**Fig. 1:**
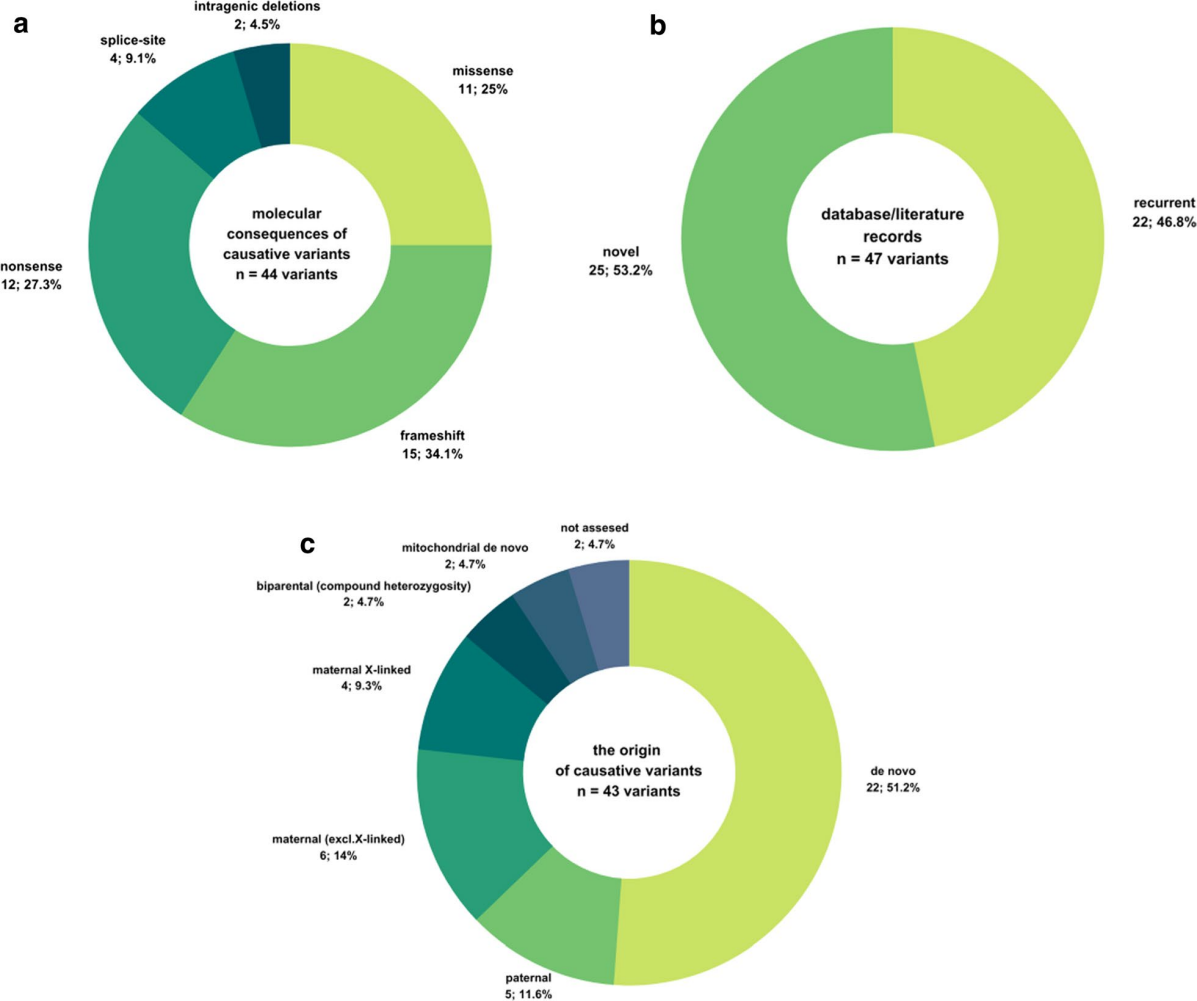
Overview of causative variants detected in this study. **a** Molecular consequences of 44 causative variants. **b** The presence of database/literature records for 47 causative variants. **c** The origin of 43 causative variants

The set of causative variants was then characterized in terms of their molecular consequences. The highest proportion was made up of truncating variants, including 34.1% (15/44) annotated as frameshift and 27.3% (12/44) of stop-gain (nonsense) variants. Other 25.0% (11/44) variants were categorized as missense, followed by 9.1% (4/44) of splice-site variants, which break a canonical donor splice site (3 cases) or acceptor splice site (1 case). Of the total number of 47 variants, 53.2% of them (25/47) were novel, while the remaining (46.8%, 22/47) have been published in the relevant scientific resources (Fig. 1a–c).

### Novel variants

The outputs of the variant prioritization led to the identification of novel rare causative variants (53.2%, 25/47) in “OMIM-morbid” genes with the clinical impact in pathogenesis of NDDs. The analytical process included their classification using the guidelines of the American College of Medical Genetics and Genomics (ACMG), in silico analysis using relevant databases and tools, searching for the relevant scientific literature, and finally, the genotype–phenotype correlation using the clinical data (Additional file 6).

#### Candidate genes

Furthermore, genome-wide approaches including ES may contribute to discovering novel candidate genes. The extensive process of the multistep in silico analysis to identify candidate genes then included: (1) The information obtained from the OMIM database related to terms such as “brain”, “central nervous system” or “development”, (2) The PANTHER™ Classification system to summarize gene ontology analysis, and (3) STRING analysis to elucidate protein–protein association networks. The pathogenicity classification of novel variants was tested using the integrated engines Franklin by Genoox (https://franklin.genoox.com) and VarSome [14]. Two novel variants *GRIN3B*(NM_138690.3):c.931C > T and *ASAP1*(NM_018482.4):c.1867C > T were prioritized (Additional files 5, 7). The additional in silico analyses using the PANTHER™ Functional classification including the candidate genes and their top 10 interaction partners were performed based on the STRING Interaction Network. The *GRIN3B* gene has been assigned in all PANTHER™ Ontologies, suggesting its important role in cellular signalling as a transmembrane signal receptor. Unlike the *GRIN3B* gene, the *ASAP1* gene has been assigned to only three of five PANTHER™ ontologies.

### Variants of borderline classification of pathogenicity

A considerable number of variants is still lacking the conclusive classification in relation to the tested genetic condition and remains on the borderline classification of pathogenicity. The prioritized variants included two variants in candidate genes (*GRIN3B* and *ASAP1*; mentioned above), five novel variants in known NDD genes (*HUWE1, KDM3B, CREBBP, TAOK1* and *PIK3R1*), a recurrent variant in the *PGK1* gene and a recurrent haplotype in the *ZGRF1* gene. Their molecular and clinical consequences of these variants are described in Additional files 5 and 7.

### Multiple-hit model and dual diagnoses

The genetic heterogeneity of NDDs and MCAs was declared by eight index cases in which more than one causative/possibly causative variant was detected, and one index case with the combination of a recurrent haplotype in the *ZGRF1* gene of paternal origin and two non-polymorphic CNVs of maternal origin. The conclusion on their clinical impact on the phenotype was done based on the comprehensive analysis of the molecular mechanism of their pathogenicity, genotype–phenotype correlation, familial segregation of the abnormal phenotypic manifestation and relevant scientific literature. Moreover, the role of the genetic background with possible epistatic interactions and other effects between the affected loci could be an underlying mechanism for the abnormal phenotypic presentation. The cases are listed in Additional file 5 and characterized in detail in Additional files 6 and 7.

### Secondary findings

Large-scale ES may also uncover P and LP variants not related to the primary diagnosis. Before the study enrolment, the parents/legal guardians were counselled about this possibility to choose to participate or not in this analysis in the informed consent [15]. SFs were found in four genes *BRCA1 (*rs80357609*,* rs80358002), *BRCA2* (rs80359351)*, HFE* (rs1800562)*, TGFBR1* (NM_004612.4:c.1133A > G) in seven individuals (3 index cases and 4 parents) (Additional file 8). None of the carriers of SFs have manifested the related conditions so far.

### Copy-number variations (CNVs)

The simultaneous detection of CNVs and genomic regions with long continuous stretches of homozygosity (LCSH) was aimed to verify ES as a compatible and complementary method for CMA and to improve the diagnostic yield beyond the scope of variant calling. An additional diagnostic yield reached 3.3% (3/90) through on the presence of a causative familial intragenic deletion in the *GRIN2A* gene and de novo deletion in the *ZC4H2* gene, both confirmed by qPCR in corresponding families (Additional file 9a). All but one non-polymorphic CNVs > 100 kb identified by CMA (96%, 26/27) in index cases were detected by ES (Additional file 9b).

### Long continuous stretches of homozygosity (LCSH)

Based on clinical information, any consanguineous families which should be revealed due to the presence of multiple LCSH were not enrolled in the study. After excluding common LCSHs and manual curation the CMA outputs and SNV/indel analysis using trio ES data, no additional LCSHs harbouring homozygous causative variants were identified. A mosaic LCSH affecting the short arm of chromosome 11 (11p) was uncovered in the index case 44-P as described in our previous study [16].

### Parental sample pooling

The parental sample pooling was tested as an alternative, cost-effective strategy for trio-based ES in the routine molecular diagnostics. De novo origin of causative variants in index cases (*MED12*, *CHD2*, *CHD*7, *IRF2BPL*, *RAI1* and *BCL11B)* as well as the familial segregation (*PBX1, CUX2, GABRB2, CACNA1C* and *NFIB* variants) was then discriminated by Sanger sequencing in corresponding families (Additional file 5). Sanger sequencing also specified the individuals in the pooled parental samples carrying P or LP variants in the *CFTR* gene, and the variant FVL (*F5* gene) due to the increased carrier population frequency.

In pooled parental samples, the average alternative allele frequencies (AAFs) of P and LP heterozygous variants (including those for human congenital disorders with AR inheritance) were calculated from the representative sample of P and LP variants. No considerable differences between observed and expected mean AAF were found (Table 1).

**Table 1:** The comparison between the observed and expected AAF in parental pooled samples

Type of pool	M/F	# of variants evaluated	Mean AAF (%)	Median AAF (%)	Minimal AAF (%)	Maximal AAF (%)	Expected mean AAF (%)
2-sample	M	15	24.53	25.00	14.89	29.41	25
2-sample	F	23	21.13	22.22	10	29.07	25
3-sample	M	18	15.14	15.90	8.7	21.43	16.67
3-sample	F	12	15.37	14.43	9.33	26.98	16.67

The varians considered for the evaluation of differences between the observed and expected AAF in parental pooled samples include familial causative variants (i.e. observed in probands and corresponding parental pool) and rare P and LP variants for autosomal recessive disorders and F5 gene FVL variant. The observed mean AAF (%) and expected mean AAF (%) are marked in bold. M—mother (maternal pool), F—father (paternal pool)

The CNV calling was initially performed in the index cases and parental pools, however, the CNV prioritization was directly done only in index cases. The familial segregation of a rare 19q13.3 microduplication (family 75) and an 18q12.1 microdeletion (family 73) was resolved using qPCR (Additional file 9a).

## Discussion

Despite the rapid progress in the development and implementation of advanced genomic analyses, the understanding of the aetiology of NDDs remains challenging due to their broad genetic and phenotypic heterogeneity. Nowadays, trio-based ES represents an effective tool to elucidate the molecular genetic diagnosis as well as to uncover novel genetic loci responsible for abnormal phenotypes. It has become an integral part of routine molecular diagnostics algorithms in a growing number of laboratories due to its clinical benefit, cost effectiveness and reduced turnaround time.

In this study, a molecular diagnosis was achieved for 44 out of 90 children with NDDs for 85 families (trios or foursomes), resulting in a total diagnostic yield of 48.9%. Pathogenic SNVs and indels were identified in 45.6% (41/90) and causative intragenic CNVs were detected in 3.3% (3/90) of affected children. Generally, trio-based ES resolves the molecular diagnosis in approximately 36% of individuals with NDDs (ranging from 31% for isolated NDDs to 53% for NDDs with associated congenital abnormalities), which greatly exceeds the 15–20% diagnostic rate for CMA [2, 17–19].

The functional analysis using PANTHER Classification system [20], which combines gene function, ontology, pathway, and statistical analysis, showed that 41 genes altered by causative variants are involved in fundamental developmental processes and cellular functioning. These functional analyses provided further evidence to the diverse phenotypic effects of causative variants, highlighting the phenotypic heterogeneity of NDDs.

De novo variants comprised the highest proportion 51.2% (22/43) of total causative variants detected by trio/foursome-based ES. They were associated with autosomal dominant, X-linked or mitochondrial inheritance for NDDs, confirming the crucial role of affected genes in the development and functioning of the CNS [21]. The loss-of-function causative variants change evolutionary conserved amino acid residues which exhibit an intolerance to variation [22]. Moreover, the genes involved in (neuro) developmental processes are strongly evolutionarily conserved to act in multiple conserved pathways [23, 24].

The familial occurrence of causative variants was uncovered in 15.6% (14/90) of paediatric patients from twelve families in which the phenotypic heterogeneity of NDDs was observed. This heterogeneity was attributed to variable combinations of de novo or familial causative variants (six families) as well as familial segregation of single causative variants with incomplete penetrance and/or variable phenotypic manifestation (six families). Other genetic and non-genetic modifiers and their epistatic interactions can modulate the phenotypic manifestation. Conversely, the dual diagnosis should be considered in cases of co-occurrence of multiple highly penetrant causative variants [23].

The prioritized variants were classified using the Franklin (https://franklin.genoox.com/) and VarSome engines [14] which integrate basic and advanced annotations, a wide variety of in silico prediction tools to obtain pathogenicity scores, population-specific allelic frequencies as well as the default final classification using the ACMG criteria. Additional in silico analyses using NMDEsc Predictor [25] and NMDetective tools [26] were performed for the prediction of the molecular consequences of PTCs. The battery of rules suggests the degradation of aberrant transcripts by NMD or their translation to an altered protein with gain-of-function or dominant-negative effects [27]. Since most PTCs were predicted to initiate the process of NMD, the haploinsufficiency of a particular gene is suggested as the leading molecular mechanism of the related condition. In common, haploinsufficiency of those genes encoding transcription factors and chromatin regulators has been suggested as a mechanism of pathogenesis for ASD and developmental disorders [28]. Another set of in silico prediction tools (Human Splicing Finder, SpliceAI and MutationTaster 2021) served to predict the molecular consequences of splicing variants [29–31]. However, not only canonical splice site variants may lead to splicing defects and deleterious molecular consequences. Cryptic splice site variants arising from deep intronic variants or apparently benign sequence changes contribute up to 11% of cases of ASD [30]. Variant classification should be perceived as a dynamic process including periodic reanalysis in the context of updates in bioinformatics, novel variant annotations and clinical data as they may be beneficial for an additional 10–15% of individuals without a conclusive diagnosis after the initial ES [32]. Moreover, complementary analyses such transcriptional profiling using RNA sequencing or methylation profiling can be beneficial for those individuals lacking a molecular diagnosis after ES or genome sequencing (GS).

Large-scale ES/GS significantly improves the diagnostic standards not only by increasing the diagnostic rate but also by detecting SFs in genes which are not related to the primary indication for the ES/GS. Reporting SFs altering “medically-actionable” genes defined by the ACMG recommendation can result in a profit due to the prevention of life-threatening conditions [15]. The observed yield of SFs, 2.7% (7/261), corresponds to an expected rate of < 3% of individuals who are commonly identified as carriers of at least one reportable SF in one of those genes defined by the ACMG. The proportion of SFs in ES/GS may vary depending on the occurrence of specific variants in founder populations [33]. Referring the SF variants to clinicians is crucial to provide an early intervention and to reduce the life-threatening effects.

The integrative ES analysis of causative sequence variants and CNVs resolves the molecular diagnosis in more than 50% of individuals with NDDs [34]. However, the reliability of CNV detection from ES data can be affected by several factors, including the design of the capture kit, sequencing depth and the choice of computational algorithms. In this study, the combination of library preparation using the Human Core Exome kit enriched by spiked-in RefSeq panel and custom spiked-in probes for mtDNA (Twist Biosciences), sequencing using the Illumina NovaSeq 6000 and two different bioinformatic pipelines for CNV detection was proved as an optimal strategy for ensuring data credibility. ES with a coverage of at least 100 × is a suitable approach for the detection of large CNVs as well as intragenic CNVs [35]. The detection of the regions of homozygosity can narrow down the number of prioritized variants and reduce the turnaround time in consanguineous families where large chromosomal segments/haplotypes are transmitted across generations. In common, the identification of multiple LCSH using SNP arrays or ES/GS can indicate a parental consanguinity, which increases the risk of homozygous causative variants and related AR phenotypes [36]. The utility of ES in offspring of consanguineous couples improves the diagnostic yield up to approximately 55% by identifying causative homozygous variants [37].

As a final step in the study, an alternative strategy involving the pooled parental samples was tested in a total of fourteen pools per two or three sex-matched parental samples. This approach shows promise as a cost-effective alternative for routine molecular diagnostics. However, the efficiency of this approach for detecting low-level mosaicism decreases as more parental samples are pooled. Even if Sanger sequencing is applied, its sensitivity in detecting somatic mosaicism is limited to 15–20%. In general, less than 5% of apparently de novo causative variants arise from low-level parental mosaicism (< 10% variant frequency in a tested tissue) [38]. The low-level somatic mosaicism occurs in both parental and maternal samples equally in contrast to a gonadal mosaicism which is strongly disproportionate and prevailing in the paternal germline due to the multiple meiotic divisions during the spermatogenesis. However, the discrimination between a true-positive low-level mosaicism from cross-contamination or background noise remains challenging so far. Therefore, the development of novel computational pipelines is strongly encouraged [39]. To detect CNVs from sequencing depth data in parental samples using the sample pool strategy is not optimal, as accurately detecting these variants in low allelic representation within the pool is methodologically challenging. Thus, verifying the causative CNVs in index and corresponding parental samples through alternative methods such as CMA, MLPA, or qPCR in parental samples, as previously suggested, is necessary [40].

## Conclusion

ES significantly improves the diagnostic yield for individuals with unexplained NDDs and associated congenital abnormalities in contrast to standard routine diagnostics approaches. It represents a credible and cost-effective tool for the simultaneous detection of DNA sequence variants and CNVs. Implementation of ES in the diagnostic algorithm can reveal novel candidate genes for NDDs and enhance our understanding of the genetic aetiology behind rare paediatric disorders of neuronal development. Finally, elucidation of the molecular mechanisms involved in the pathogenesis of NDDs would improve genetic counselling, leading to the prevention of medical complications and better utilization of supportive resources.

## Methods

### Patient recruitment and sampling

The informed consent for this study has been approved by the Research Ethics Committee of Masaryk University and Ethics Committee of University Hospital Brno. The risk of secondary findings (SF) and their clinical impact has been fully explained by the clinical geneticists The legal guardians have been asked to opt in or opt out to receiving SF. Their interpretation and related genetic counselling have been provided by the clinical geneticists.

Totally 90 paediatric patients (index cases) from 85 families including 79 trios (affected individuals with parents, 78 cases; or the affected individual and unaffected parent and sibling, one case) and six foursomes (affected siblings with parents, five cases; affected individual with parents and unaffected sibling, one case) were recruited at the Department of The Medical Genetics and Genomics (University Hospital Brno) from May 2020 to December 2022. The index cases were evaluated clinically with inclusion criteria: unexplained severe neurodevelopmental disorder (intellectual disability, autism spectrum disorder or global developmental delay) with possible multiple congenital abnormalities. The age profile of the study cohort and clinical information are summarized in Tables 2 and 3.

**Table 2:** The age profile of the study cohort

	# of individuals	Median age (years)	Minimum age (years)	Maximum age (years)
		Age in the year of recruitment		
Index cases	90	7	0	34
		Age at conception/delivery		
Mothers	85	31	19	41
		Age at conception/delivery		
Fathers	84	33	20	52

**Table 3:**
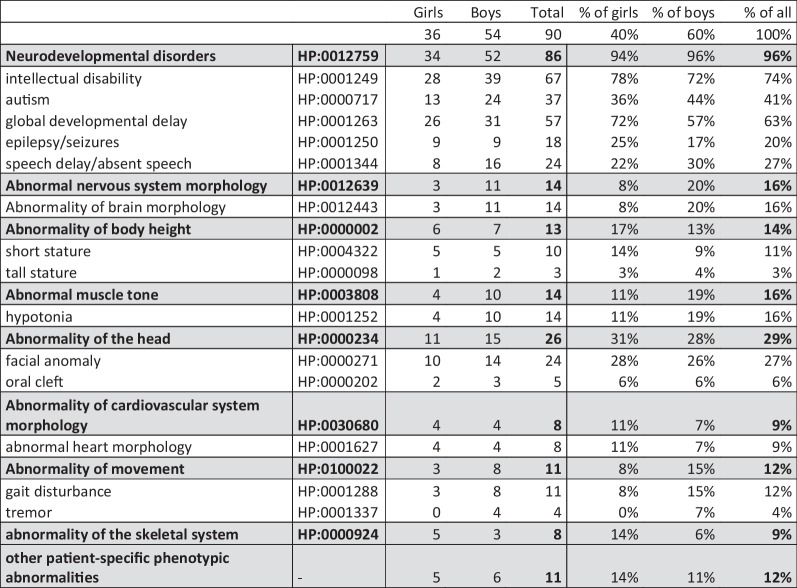
Summary of paediatric patients' clinical records

Phenotype/HPO (grey background) represents a collective term. The absolute counts and % (bold in grey background) summarize that at least of one included phenotype/HPO term (white background) has been observed in the index case

The routine cytogenetic analysis of a karyotype and chromosomal microarray analysis (CMA) [41] using CGH or CGH + SNP arrays without conclusive molecular diagnosis preceded ES. Peripheral blood samples were collected in sterile heparinized tubes for cytogenetic analysis. Genomic DNA samples were extracted from 1 ml of peripheral blood using the MagNaPure system (Roche Diagnostics, Basel, Switzerland), LabTurbo Compact System (LabTurbo, Shilin Dist., Taipei City, Taiwan) or phenol–chloroform extraction. Quality control metrics were then assessed using the NanoDrop® ND-1000 (Thermo Fisher Scientific, Inc., Waltham, MA, USA) and the Qubit® 2.0 Fluorometer (Thermo Fisher Scientific, Inc.). The cytogenetic analysis of the karyotype was performed using a routine G-banding procedure, followed by CMA using SurePrint G3 CGH and CGH + SNP Microarray platforms (Agilent Technologies, Inc., Santa Clara, CA, USA), according to the manufacturer's recommendations as described elsewhere [42]. Moreover, the study excluded those cases which were concluded as Fragile X syndrome or those which were elucidated by molecular genetic testing (small- or medium-sized “next generation” sequencing panels or Sanger sequencing).

Before the enrolment in the trio ES, the legal guardians (parents) signed an informed consent (approved by the Research Ethics Committee of Masaryk University and Ethics Committee of University Hospital Brno).

### Exome sequencing

High-quality genomic DNA samples of required quantities were used for a library preparation with the Human Core Exome Kit enriched by spiked-in RefSeq panel (Twist Bioscience, San Francisco, CA, USA) and custom spiked-in probes for mtDNA. DNA libraries were then sequenced on the Illumina NovaSeq 6000 (Illumina, Inc., San Diego, CA, USA). The steps of DNA samples processing and sequencing were purchased as a commercially available service (Institute of Applied Biotechnologies, Olomouc, Czech Republic).

In the final phase of the study, a subset of parental DNA samples was proposed to test and verify the design of pooled parental samples as suggested before [40]. DNA samples of seventeen index cases were processed to libraries and sequenced as mentioned above. The parental samples were precisely quantified and diluted, if necessary. Their equimolar amounts were mixed to get eight independent pools: four pools per two maternal samples, four pools per two paternal samples, three pools per three maternal samples and three pools per three paternal samples. Then the pooled samples were processed to libraries and sequenced.

### Bioinformatics processing of ES data

Raw sequencing data were processed as described elsewhere [43, 44]. Briefly, the quality control (QC) was performed using the FastQC v0.11.9 (released 8th January 2019; https://www.bioinformatics.babraham.ac.uk/projects/fastqc/) and by Picard v2.25.6 (released 15th June 2021; https://broadinstitute.github.io/picard/). After the low-quality reads and adapter contamination trimming by the fastp v0.20.1 (released 8th April 2020; https://github.com/OpenGene/fastp/tree/v0.20.1) the remaining reads were aligned to the reference human genome hg38 primary assembly by BWA v0.7.17-r1188 with default parameters (released 23rd October 2017; https://github.com/lh3/bwa/tree/v0.7.17). Marking duplicate reads and fix mate information was then performed by Picard Toolkit (http://broadinstitute.github.io/picard/). QC steps and the coverage were reviewed using the in-house software Genovesa (Bioxsys, s.r.o., Czech Republic). The single nucleotide variant (SNV) and CNV calling with further prioritization were described previously [43, 44] and in the Additional file 10.

In the parental sample pooling design, VCF files of index cases and corresponding parental pools (containing parental samples for the given index case) were merged to streamline the variant prioritization and to assess the parental segregation of familial variants. The heterozygous calls in pooled samples were expected to be in lower proportions of reads than observed in non-pooled samples. The expected percentage of carrying the heterozygous call in a pool (N) was calculated using the formula $N=\frac{1}{\left(n parents x 2 alleles\right)}\times 100\%$ [40]. With a uniform enrichment of targeted regions and a median sequencing depth of 100X, an average heterozygous call in 25% (two samples per pool) and 16.67% (three samples per pool) of mapped reads was expected, respectively.

The pooled parental samples were added to index cases to normalize read counts to produce the pooled reference for CNV calling. Therefore, the CNVs were called by only the optimized *in-house* bioinformatics pipeline (using Genovesa), prioritized only in index cases, and defined by technical thresholds with reads ratio ≤ 0.7 for losses and ≥ 1.3 for gains.

### *Gene-set *in silico* analysis*

The in silico analysis using PANTHER™ Classification system v17.0 (released 23rd February 2022; http://www.pantherdb.org/) [20] was used for the functional classification of gene set with reported causative (pathogenic and likely pathogenic; P and LP) variants. The gene set was then loaded into the web interface for the statistical overrepresentation test (with false discovery rate, FDR, p < 0.05).

The gene interactions were then studied using the STRING Interaction Network v11.5 (released 12th August 2021; https://string-db.org/) for candidate genes with novel variants [45]. First, the analysis was run to specify top 10 predicted interaction partners with the default settings as follows: Network Type: full String interaction network; Required score: medium confidence (0.400); Size cut-off: no more than 10 interactors. The candidate gene and its top 10 predicted interaction partners were loaded into the web interface for in silico analysis using PANTHER™ Classification system with default setting for both functional classification and statistical overrepresentation test. The overrepresentation test was performed against the reference list represented by all genes in the database for Homo sapiens (20,589 genes). Only outputs with FDR *p* < 0.05 were considered for further evaluation.

### Sanger sequencing

After the variant prioritization, clinically relevant sequence variants (de novo*,* or inherited) were verified using Sanger sequencing as described elsewhere [43]. Sanger sequencing also served for the determination of the sample carrying P or LP variants including SF in parental sample pools.

### Quantitative real-time PCR (qPCR)

The non-polymorphic and/or clinically relevant CNVs that were below the detection limit of microarray analysis were verified using qPCR with custom-designed primers as described elsewhere [42]. The reactions were run in duplicates for the index case, parents, and a commercially available reference DNA sample (Agilent Technologies) using the Power SYBR Green PCR Master Mix and default cycling conditions following the manufacturer’s recommendations (Thermo Fisher Scientific). The *ERH* gene served as an endogenous control. The relative quantification was assessed from C_t_ values using the calculation of R-values (R = 2^−ΔΔCt^). The R-value cut-offs were set at < 0.7 for DNA losses and > 1.3 for DNA gains [46].

### Supplementary Information

**Additional file 1: **Quality Control (QC) metrics for outputs from exome sequencing.

**Additional file 2: **Functional Classification Analysis using the PANTHER^TM^ Classification system.

**Additional file 3: **The Gene Ontology (GO) analysis using the PANTHER^TM^ Classification system.

**Additional file 4: **PANTHER Overrepresentation Test for the gene set with causative variants.

**Additional file 5: **Molecular characterization of causative variants and variants with borderline classification of pathogenicity.

**Additional file 6: **Molecular characterization of novel candidate variants in NDD genes and novel variants in the candidate genes and their clinical consequences.

**Additional file 7: **Molecular characterization of variants with borderline classification of pathogenicity (VUS-LP) and their clinical consequences.

**Additional file 8: **Molecular characterization of secondary findings in the “medically-actionable” genes on the ACMG list.

**Additional file 9: **a) Non-polymorphic intragenic CNVs detected by ES, b) Non-polymorphic CNVs initially detected by CMA and then verified by ES.

**Additional file 10: **The SNV and CNV detection and prioritization.

## Data Availability

The datasets generated and analysed during the study are not publicly available to protect personal data privacy but are available from the corresponding author on reasonable request. All novel causative and candidate sequence variants were submitted to the Global Variome shared LOVD v.3.0—Leiden Open Variation Database (https://www.lovd.nl/) (see Additional file 5 for their IDs). The detailed outputs from the STRING Interaction Network and PANTHER™ Classification system (Functional analysis and Overrepresentation analysis) for interaction networks including candidate genes *GRIN3B* and *ASAP1* are available on the Figshare Online Repository, doi: 10.6084/m9.figshare.22300378.

## Abbreviations

AAF: Alternative allele frequency
ACMG: American College of Medical Genetics and Genomics
AD: Autosomal dominant
AR: Autosomal recessive
ASD: Autism spectrum disorders
CMA: Chromosomal microarray analysis
CNVs: Copy-number variations
CNS: Central nervous system
ES: Exome sequencing
GO: Gene Ontology
GS: Genome sequencing
LCSH: Long continuous stretches of homozygosity
LP: Likely pathogenic
MCAs: Multiple congenital abnormalities (MCAs)
MLPA: Multiplex ligation-dependent probe amplification
NDDs: Neurodevelopmental disorders
P: Pathogenic
PTC: Premature termination codon
QC: Quality control
qPCR: Quantitative real-time PCR
SF: Secondary finding
VUS: Variant of uncertain significance
