# Supplementary material for: Exome sequencing improves the molecular diagnostics of paediatric unexplained neurodevelopmental disorders
Source: Orphanet J Rare Dis. 2024 Feb 6;19:41. doi: 10.1186/s13023-024-03056-6 (PMC10845791; doi:10.1186/s13023-024-03056-6)
Supplement: Supplementary file 1 — Additional file 1: Quality Control (QC) metrics for outputs from exome sequencing. [file 13023_2024_3056_MOESM1_ESM.docx]

**Additional File 1: Quality Control (QC) metrics for outputs from exome sequencing**

1. The quality control (QC) was done using the FastQC v0.11.9 on 241 samples. The 14 pools of parental samples were considered as 14 individual samples. The definitions of the QC metrics were obtained from Picard Metrics Definitions: HsMetrics. Available on <https://broadinstitute.github.io/picard/picard-metric-definitions.html> [2023-03-17]


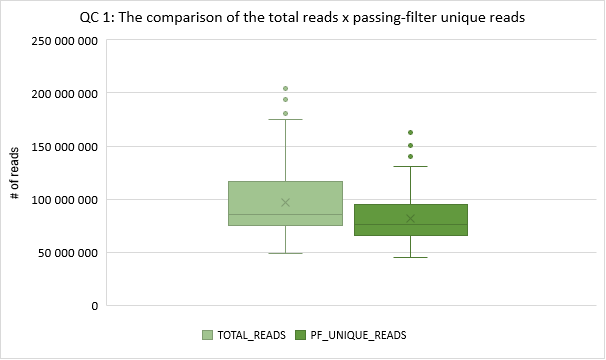


**Figure S1a**: The box-plot comparison of the total number of reads in the examined SAM/BAM files (TOTAL_READS) and the number of passing-filter non-duplicated reads (PF_UNIQUE_READS).

**Figure S1b:** The comparison of total number of reads in the SAM/BAM files examined (TOTAL_READS) and the number of passing-filter non-duplicated reads (PF_UNIQUE_READS).

**Figure S1c**: The proportion of the fraction of passing-filter aligned bases* located on or near a baited region (PCT_SELECTED_BASES) and the fraction of passing-filter aligned bases* that are mapped away from any baited region (PCT_OFF_BAIT).

*Passing-filter aligned bases = The number of passing-filter unique bases that are aligned to the reference genome hg38/GRCh38 primary assembly with mapping scores > 0.


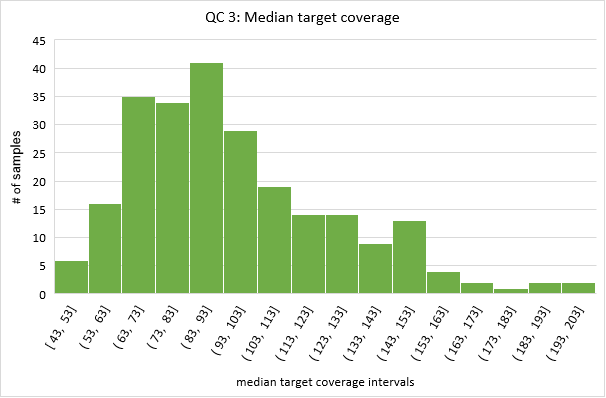


**Figure S1d:** The median coverage of a target region. The interval range is 10 units. The average median value was calculated as 97X.


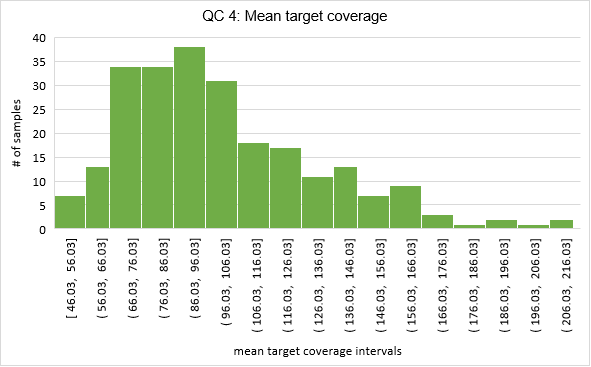


**Figure S1e:** The mean coverage of a target region. The interval range is 10 units (samples). The average mean value was calculated as 102X.


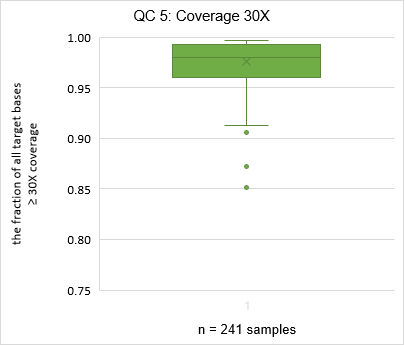


**Figure S1f:** The fraction of all target bases achieving 30X or greater coverage. The average value was calculated as 98% of all target bases. Only two samples had less than 90% of all target bases (85% and 87%) achieving ≥30X coverage.


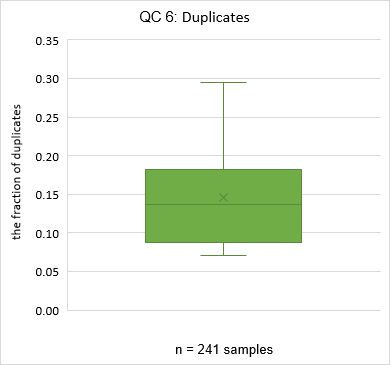


**Figure S1g:** The box-plot visualization of the fraction of aligned bases that were filtered out because they were in reads marked as duplicates. The average value was calculated as 0.15 (15%).

1. The quality control (QC) was done using the FastQC v0.11.9 on 18 index cases and 14 corresponding pooled parental samples. The 14 pools of parental samples were considered as 14 samples for QC metrics evaluation. The definitions of the QC metrics were obtained from Picard Metrics Definitions: HsMetrics. Available on <https://broadinstitute.github.io/picard/picard-metric-definitions.html> [2023-03-17]


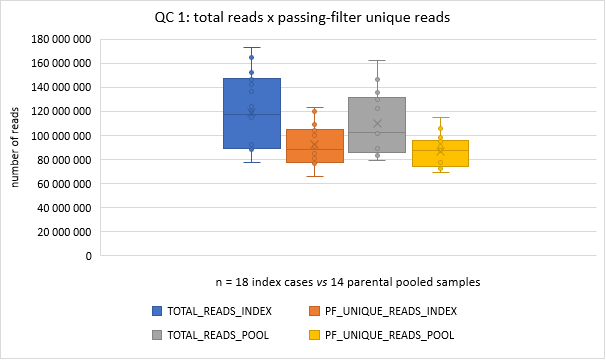


**Figure S1**: The box-plot comparison of total number of reads in the SAM/BAM files examined (TOTAL_READS) and the number of passing-filter non-duplicated reads (PF_UNIQUE_READS). Outputs were generated for 18 index cases (INDEX) and 14 corresponding pooled parental samples (POOL). Average values are marked as crosses (x) in box-plots.

**Figure S2:** The comparison of total number of reads in the SAM/BAM files examined (TOTAL_READS) and the number of passing-filter non-duplicated reads (PF_UNIQUE_READS). Outputs were separately generated and visualized for 18 index cases (QC 2a) and 14 corresponding pooled parental samples (QC 2b).

**Figure S3**: The proportion of the fraction of passing-filter aligned bases* located on or near a baited region (PCT_SELECTED_BASES) and the fraction of passing-filter aligned bases* that are mapped away from any baited region (PCT_OFF_BAIT). The outputs are summarized for 18 index cases and corresponding 14 parental pooled samples.

*Passing-filter aligned bases = The number of passing-filter unique bases that are aligned to the reference genome hg38/GRCh38 primary assembly with mapping scores > 0.


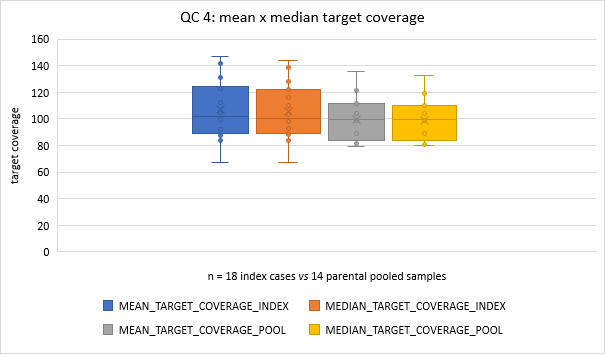


**Figure S4:** The mean and median coverage of a target region in index cases (n = 18) and corresponding parental pooled samples (n = 14). The interval range for target coverage is 20 units. For index cases, the average mean target coverage was calculated as 107X and the average median target coverage as 105X, respectively. For parental pooled samples, the average mean target coverage was calculated as 100X and the average median target coverage as 99X, respectively. Average median and mean values are marked as crosses (x) in box-plots.
