# Supplementary material for: Exome sequencing improves the molecular diagnostics of paediatric unexplained neurodevelopmental disorders
Source: Orphanet J Rare Dis. 2024 Feb 6;19:41. doi: 10.1186/s13023-024-03056-6 (PMC10845791; doi:10.1186/s13023-024-03056-6)
Supplement: Supplementary file 2 — Additional file 2: Functional Classification Analysis using the PANTHERTM Classification system. [file 13023_2024_3056_MOESM2_ESM.docx]

**Additional File 2: Functional Classification Analysis using the PANTHER^TM^ Classification system.**

PANTHER^TM^ GO-slim uses a selected set of terms from the Gene Ontology ^TM^ (GO) for classifications by molecular function, biological process, and cellular component. The PANTHER^TM^ Protein Class ontology was adapted from the PANTHER/X molecular function ontology, and includes commonly used classes of protein functions, many of which are not covered by GO molecular function.

Molecular function is the function that a protein performs on its direct molecular targets. Cellular component is the location where the protein performs its molecular function. Biological process covers the biological systems to which a protein contributes. PANTHER^TM^ annotations are more reliable the GO annotations since they are manually curated. PANTHER^TM^ uses only a subset of GO terms (“GO slim”) to facilitate browsing.

Retrieved from: <http://www.pantherdb.org/panther/ontologies.jsp> [2023-02-05]; modified.

PANTHER™ GO slim includes 3362 total terms, 2267 biological process terms, 544 component terms, 550 molecular function terms. PANTHER^TM^ Protein Class includes 209 total terms. PANTHER^TM^ Pathway 3.6.6 (released 2022-02-22) includes 177 pathways, 3092 pathway components, 51914 sequence associated to pathways, 5996 references captured for the pathways.

Retrieved from: <http://www.pantherdb.org/data/>, modified. For more detailed information please visit: <http://www.pantherdb.org/dataFlow.jsp>

The PANTHER^TM^ (version 17.0, based on Gene Ontology release 2021-11-16, released 2022-02-22) data types, Pathway (PANTHER^TM^ Pathway) and Ontologies (PANTHER^TM^ GO-Slim and PANTHER^TM^ Protein Class) were selected for the functional classification analysis of the set of 41 genes in which causative (P and LP) variants for paediatric NDDs were detected. The graphical outputs for ontologies PANTHER^TM^ GO-Slim Molecular Function and Biological process are listed below. The detailed outputs including the categorization of genes from the set of 41 genes analysed are available in **Additional File 3** (the sheets according to the type of the ontology).

The outputs were checked and orthologs were removed: GRIN1 (PTN002557551; G protein-regulated inducer of neurite outgrowth 1) and CHD2 (DSCAM PTN002543450; Down syndrome cell adhesion molecule).

**
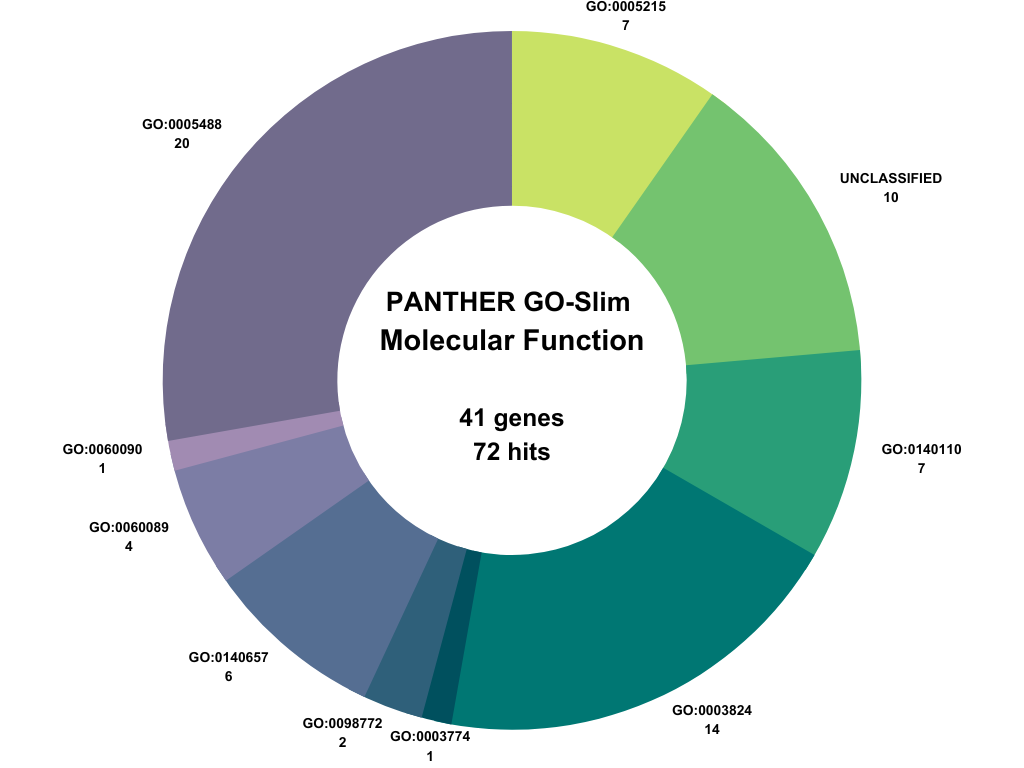
**

**Figure S5a: The graphical output for the Gene Ontology PANTHER GO-Slim Molecular Function**. The categories are marked by GO terms (accession code). The fractions represent the number of genes (out of 41) with causative variants in the corresponding category, out of the total number of Function hits (72).

**
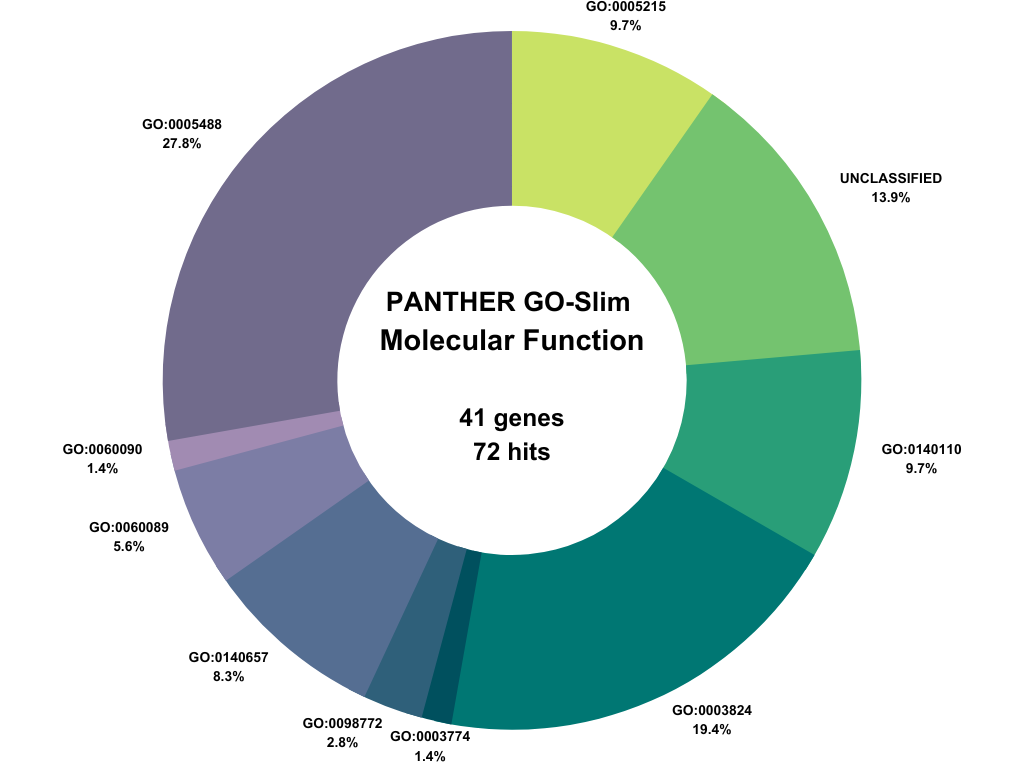
**

**Figure S5b: The graphical output for the Gene Ontology PANTHER GO-Slim Molecular Function**. The categories are marked by GO terms (accession code). The fractions represent the % of genes (out of 41) with causative variants in the corresponding category, out of the total number of Function hits (72).

**
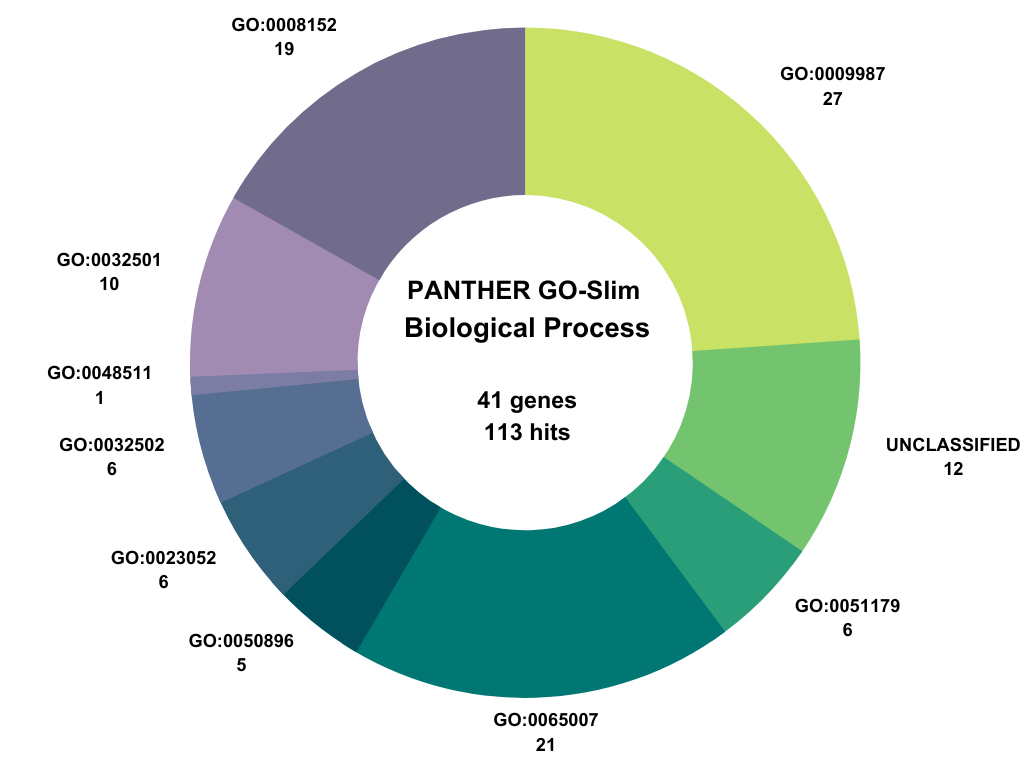
**

**Figure S5c: The graphical output for the Gene Ontology PANTHER GO-Slim Biological Process**. The categories are marked by GO terms (accession code). The fractions represent the number of genes (out of 41) with causative variants in the corresponding category, out of the total number of Process hits (113).

**
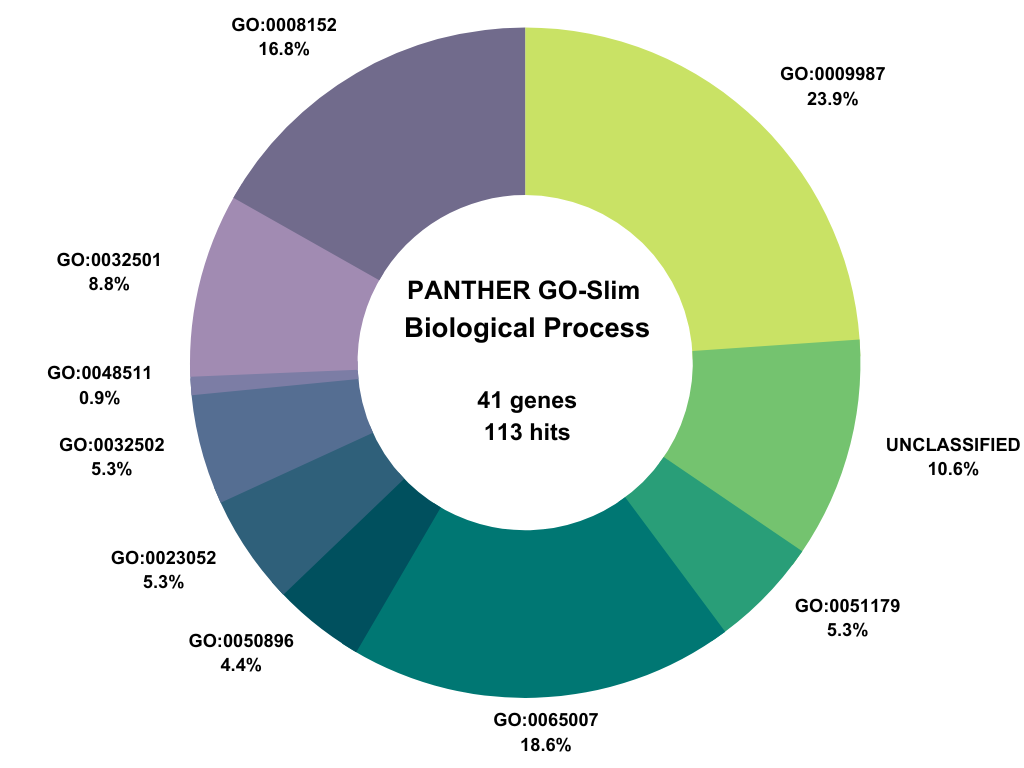
**

**Figure S5d: The graphical output for the Gene Ontology PANTHER GO-Slim Biological Process**. The categories are marked by GO terms (accession code). The fractions represents the % of genes (out of 41) with causative variants in the corresponding category, out of the total number of Process hits (113).
