# Supplementary material for: Exome sequencing improves the molecular diagnostics of paediatric unexplained neurodevelopmental disorders
Source: Orphanet J Rare Dis. 2024 Feb 6;19:41. doi: 10.1186/s13023-024-03056-6 (PMC10845791; doi:10.1186/s13023-024-03056-6)
Supplement: Supplementary file 6 — Additional file 6: Molecular characterization of novel candidate variants in NDD genes and novel variants in the candidate genes and their clinical consequences. [file 13023_2024_3056_MOESM6_ESM.docx]

# **Additional File 6: Molecular characterization of novel candidate variants in NDD genes and novel variants in the candidate genes and their clinical consequences**

The summary of the description using the HGVS nomenclature as well as *in silico* predictions of the molecular consequences are available in the Additional File 5.

## **(A) Novel variants in NDD genes**

**A.1. Missense variants**

***GNAI1***

A novel variant in the *GNAI1* gene, p.(Asp272Gly) (NM_002069.6:c.815A>G) was identified in monozygotic twins (*2007) with severe global developmental delay. This variant results in an amino-acid substitution of aspartic acid to glycine in the GTP/GDP binding region (aa positions 269-272) and was described recently [1].

***DYNC1H1***

A familial pathogenic variant of the *DYNC1H1* gene (NM_001376.5:c.7059G>C) was found in the index case 32-P with severe psychomotor delay, developmental dysphasia, dyspraxia and attention-deficit disorder, while a low-level mosaicism was detected in peripheral blood (~15%) and buccal swab (~10%) of her father 32-F with a subtle specific learning disorder. The variant affects the AAA2 motor domain of the protein, explaining the phenotypic manifestation of symptoms in both the index case and her father [2].

***SMARCA2***

A *de novo* variant NM_003070.5:c.3673G>A in the *SMARCA2* gene was identified in the index case 33-P with severe intellectual impairment, abnormal facial appearance and 5^th^ finger clinodactyly. It is located downstream the Helicase C-terminal domain and outside the functional domains of which alterations are responsible for the manifestation of Nicolaides-Baraitser syndrome (NCBRS) (ATPase domain or nearby residues) or blepharophimosis intellectual disability syndrome (BIS) (clusters in exons 8, 9, or 19 outside the ATPase helicase domain). Further specialized clinical examination could either specify the syndrome or evaluate the clinical manifestation of symptoms as non-syndromic [3].

***NALCN***

A missense variant NM_052867.4:c.4073G>A was identified in the compound heterozygosity *in trans* with a nonsense causative variant c.2524C>T (rs779930597) in the *NALCN* gene. The missense variant c.4073G>A, p.(Gly1358Asp) is located within the extracellular topological domain in the close proximity of the transmembrane segment S5 in the pore forming domain IV (Uniprot entry Q8IZF0) and is predicted as deleterious (ACMG criteria PM2, PM3, PP2, PP3). Therefore, the compound heterozygosity for these *NALCN* gene variants explains the severe phenotypic features (severe intellectual impairment and developmental delay) observed in the index case 59-P [4].

***ITPR1***

A *de novo* missense variant NM_001378452.1:c.4205C>G within the exon 33 of the *ITPR1* gene was detected in the index case 66-P with intellectual impairment, gait abnormalities and abnormal facial appearance. The variant c.4205C>G alters neither any structural nor functional domain, however, it is proven as highly evolutionarily conserved (PhastCons score 1.0, phyloP100 score 7.842). Rare heterozygous variants of the *ITPR1* gene are responsible for the clinical manifestation of spinocerebellar ataxias (types 15 and 29) and some cases of Gillespie syndrome [5; 6].

***GABRB2***

A familial missense variant NM_001371727.1:c.869C>T within the exon 8 of the *GABRB2* gene was proven as the molecular cause of abnormal phenotypic features (global developmental delay, speech delay, hyperactivity and EEG abnormalities) in the index case 69-P and her mother 69-M. Complementary Sanger sequencing revealed this variant in other affected siblings (one brother and one sister) with similar phenotypic manifestation. At the protein level, the variant p.Thr290Ile disturbs the transmembrane helical domain (p.Ala270 to p.Leu292). The reported causative variants in the cluster spanning from position 244 to 304 are mostly associated with global developmental delay and intellectual impairment [7].

***EDA***

A novel hemizygous variant in the X-linked *EDA* gene (NM_001399.5:c.557G>A) of maternal origin was found in the index case 8-P, explaining his phenotype of anhidrotic ectodermal dysplasia and oligodontia [8]. The maternal grandfather manifests a similar phenotype (ectodermal dysplasia and oligodontia with an unspecified time of onset), but his DNA sample was not available for testing. Additionally, CMA of the index case 8-P identified a rare 2q14.3 microduplication of paternal origin encompassing the *CNTNAP5* gene, classified as VUS. Therefore, its contribution to the other phenotypic features of speech delay and autistic features cannot be excluded [9].

**A.2. Splicing variants**

*In silico* predictions using SpliceAI, Human Splicing Finder (HSF) and CADD confirmed the deleterious effect on pre-mRNA splicing for both *de novo* variants in *SHANK3* and *KDM1A* genes, the paternal variant in the *CACNA1C* gene, and the *CPA6* gene variant of unknown origin.

Two cases of children with *de novo* splice donor variants in the *KDM1A* gene (novel variant) and *SHANK3* (recurrent variant) and one case of a novel splice donor variant in the *CACNA1C* gene of a paternal origin were identified*.* The novel acceptor splice site variant NM_020361.5:c.117-2A>G in the *CPA6* gene (origin unknown) was detected in the index case 65-P.

***CPA6***

The *in silico* SpliceAI and HSF tools predicted a damaging effect lying in the loss of the acceptor splice site or the activation of the cryptic donor splice site for the splice acceptor variant in the *CPA6* gene (NM_020361.5:c.117-2A>G) observed in the index case 65-P. However, its origin is unknown due to the absence of paternal DNA sample for testing (mother and brother are negative) and therefore, suggested molecular consequences cannot be resolved in the autosomal dominant or recessive manner. Furthermore, the abnormal facial appearance observed in the index case 65-P was concluded as the typical feature of Treacher-Collins syndrome (TCS) due to the recurrent causative variant NM_001371623.1:c.4221dup (rs1561540623) in the *TCOF1* gene. Approximately 60% of TCS cases arise as the consequence of *de novo* causative variants [10].

***KDM1A***

A novel *de novo* splicing variant broke a canonical donor splice site on the borderline of exon 12 and intron 12 (NM_001009999.3:c.1413+1G>A) of the *KDM1A* gene. The *KDM1A* gene (OMIM *609132) encodes a lysine demethylase of H3K4, causing gene repression. Pathogenic variants of the gene lead to the manifestation of severe psychomotor delay, cleft palate, and distinct facial abnormalities. The KDM1A protein targets regulation sites of genes known in the pathogenesis of neurodevelopmental disorders [11]. The haploinsufficiency of the *KDM1A* and disrupted regulation of epilepsy-related genes may result in the epilepsy and epileptic syndromes as observed in the index case 4-P.

***SHANK3***

A *de novo* splice donor variant on the borderline of exon 22 and intron 22 of the *SHANK3* gene (NM_001372044.2:c.2451+1G>A) was detected in the index case 49-P affected with ASD and intellectual impairment. The haploinsufficiency of the *SHANK3* gene is a known mechanism for the phenotype of Phelan-McDermid/22q13 deletion syndrome (OMIM #606232) [ 12; 13].

***CACNA1C***

A familial variant breaking the splice donor site of the *CACNA1C* gene (NM_000719.7:c.4623+1G>A) was found in the index case 83-P. Both the index case and his father 83-F manifest the associated phenotypes (moderate intellectual impairment and facial abnormality) [14]. Moreover, another causative nonsense *NFIB* gene variant of maternal origin was found in the index case 83-P and was concluded to be responsible for the phenotypic abnormalities in the index case and his affected mother 83-M (intellectual impairment and facial abnormalities). The index case 83-P manifests the most severe phenotypic abnormalities of all affected family members. *In silico* analysis using NMDetective predicted the degradation of the abnormal *NFIB* mRNA due to the premature termination codon (PTC) by the process of nonsense-mediated mRNA decay (NMD). The haploinsufficiency of the gene *NFIB* is associated with intellectual impairment, behavioural abnormalities, and abnormal facial appearance [15].

**A.3. Protein-truncating variants (frameshift or nonsense)**

The highest proportion of novel causative variant was shown for protein-truncating variants (PTVs) including frameshift and nonsense variants (59.0%, 26/44). The accuracy of *in silico* predictions for NMD efficiency due to the presence of PTC is controversial, depending on the algorithm applying the set of specific rules for NMD in a particular *in silico* tool [16]. The MutationTaster 2021 was used for the NMD prediction in all truncating variants, NMDEsc Predictor only for frameshift variants (15 variants) and NMDetective only for nonsense variants (12 variants). NMDEsc Predictor did not generate any outputs for the 1-bp deletion variant NM_024496.4:c.508del in the intronless *IRF2BPL* gene. The positions of most PTCs were predicted as a signal for the process of NMD and degradation of abnormal transcripts.

***CHD8***

A 2-bp deletion affecting the exon 3 of the *CHD8* gene (NM_001170629.2:c.1123_1124del) was concluded to be causative for the manifestation of global developmental delay, autistic features and tall stature in the index case 37-P. The encoded protein provides a transcriptional regulation of beta-catenin target genes. The *CHD8* haploinsufficiency was described in the pathogenesis of ASD due to the impaired axon development and migration of cortical neurons [17; 18].

***KMT2E***

A *de novo* 17-bp deletion disrupting the exon 17 of the *KMT2E* gene was detected in the index case 12-P1 with a severe delay of intellectual development, ASD and behavioural abnormalities. The variant does not affect any structurally or functionally important protein domain. However, due to the production of aberrant transcripts and their degradation by NMD, it is consistent with the *KTM2E* gene haploinsufficiency and the associated O’Donnell-Luria-Rodan syndrome [19].

***RAI1***

A *de novo* deletion variant c.2526_2545del in the exon 3 of the *RAI1* gene (OMIM *607642) was found in the index case 35-P manifesting a profound intellectual impairment, obesity, visual and hearing impairment, and neurobehavioral disorder. Another *de novo* nonsense mutation NM_030665.4:c.13C>T in the exon 1 was lately revealed in the index case 82-P sharing some severe phenotypic abnormalities, including developmental and growth delay, microcephaly, hearing impairment and ichthyosis vulgaris. The haploinsufficiency of the *RAI1* gene is confirmed to play a critical role in the pathogenesis of Smith-Magenis syndrome [20; 21].

***CTNNB1***

A 1-bp insertion to the exon 6 of the *CTNNB1* gene (NM_001904.4:c.911dup) was proven in the index case 39-P with severe developmental regression, developmental delay, and intellectual impairment (“Rett-like” features). Trio-based ES uncovered a low-level mosaicism (~10%) in the peripheral blood of her unaffected father. The low-level mosaicism (~13%) was then confirmed in the DNA sample from the buccal swab. *In silico* analysis indicated that the aberrant transcripts are degraded by NMD, explaining the *CTNNB1* haploinsufficiency phenotype [22].

***DYRK1A***

A *de novo* 1-bp insertion the NM_001347721.2:c.539dup was identified in the exon 6 of the *DYRK1A* gene (OMIM *600855) in the index case 55-P affected by moderate neurodevelopmental disorder, pulmonary stenosis and abnormal facial appearance. The *DYRK1A* gene is one of the best-known gene of which truncating variants are responsible for autosomal dominant syndromic intellectual disability with microcephaly and epilepsy [23].

***IRF2BPL***

A pathogenic variants in the *IRF2BPL* gene have been reported in only a limited number of individuals with progressive early-onset neurodegeneration with impaired cognitive functions [24]. The index case 77-P was uncovered to be a carrier of a novel *de novo* 1-bp deletion NM_024496.4:c.508del. Since the *IRF2BPL* gene consists of only one coding exon, the frameshift variant results in the production of a truncated protein. The dominant-negative fashion of truncating variants has been suggested as the molecular mechanism of recently defined *IRF2BPL*-related disorders [25].

***OPHN1***

A hemizygous X-linked variant in the *OPHN1* gene, NM_002547.3c.835del, was detected in the index case 41-P. The absence of the encoded protein in males results in the manifestation of hypotonia, neurometabolic disorder and severe psychomotor delay. Pathogenic variants altering the *OPHN1* gene result in the Billuart-type X-linked syndromic intellectual development disorder [26].

***PTCHD1***

A familial X-linked variant in the *PTCHD1* gene, NM_173495.3:c.1765G>T, was detected in two index cases, 58-P1 and 58-P2, and their unaffected mother 58-M. Due to the presence of PTC, the degradation of altered mRNA transcripts by NMD is predicted. Furthermore, the index case 58-P2 is a carrier of a heterozygous variant NM_001127222.2:c.3411dup (rs746790849) in the *CACNA1A* gene of the paternal origin and a reported frequency of 1.14x10^-4^ (non-Finnish European population). The associated episodic ataxia type 2 is a neurological disorder with a variable clinical presentation and typical onset of symptoms in the second decade of life [27]. However, early-onset attacks have been observed in rare cases, and therefore, this additional causative variant in the index case 58-P2 could explain variable clinical presentation of two siblings 58-P1 and 58-P2 with X-linked familial *PTCHD1* causative variant.

***PBX1***

The family history of an abnormal phenotype characterized by intellectual impairment, motor delay and outer ear abnormalities were linked to a novel familial variant NM_002585.4:c.649C>T in the *PBX1* gene. This variant was identified in the index case 71-P and his mother 71-M. Most of the phenotypic abnormalities disappeared in the mother during puberty, except for the outer ear abnormalities. These observations are consistent with a pronounced pleiotropy of *PBX1*-related disorders [28; 29].

***CUX2***

The familial segregation of a novel *CUX2* variant NM_015267.4:c.3065C>A was proposed as a molecular cause of the intellectual impairment, autism, speech delay and hyperactivity, which were observed in the index case 70-P. The variant was inherited from a slightly affected mother with transient, early-onset epilepsy, speech disorder and mild learning disability. However, only one *de novo* nonsense variant of the *CUX2* gene has been identified in the proband with autistic features so far [30].

***BLC11B***

Unlike sporadic nonsense variants in the *CUX2* gene and their clinical implication, the truncating variants in the *BCL11B* gene have been recently described in the pathogenesis of paediatric NDDs, specifically causing intellectual impairment, facial abnormalities, speech delay and T-cell abnormalities f31]. The phenotypic overlap with this condition was observed in the index case 85-P with a novel *de novo* variant NM_138576.4:c.2037del. As the variant occurs in the last exon of the *BCL11B* gene, the presence of PTC likely does not activate the NMD pathway and therefore, the truncated protein may be produced and act in the dominant-negative fashion [32].

***MEIS2***

Pathogenic loss-of -function variants of the *MEIS2* gene have been previously implicated in the well-defined phenotypic spectrum of intellectual impairment, cleft palate, and cardiac defects [33; 34]. A novel *de novo* variant in the exon 10 of the *MEIS2* gene (NM_170675.5: c.1021C>T) was uncovered in the index case 23-P with a consistent phenotype of ventricular septal defect, soft cleft palate and developmental delay.

## **(B) Copy-number variations (CNVs)**

A familial deletion of the exon 2 of the *GRIN2A* gene (NM_001134408) was detected in siblings 43-P1 and 43-P2 with epilepsy, delayed speech and language development and behavioural abnormalities. ES uncovered its paternal origin when the father 43-F manifests subtle clinical features such as dyslexia and transient, childhood-onset epilepsy. The *GRIN2A* gene encodes a subunit of the N-methyl-D-aspartate (NMDA) receptor on excitatory synapses in the brain. The heterozygous deletion of the exon 2 leads to a complete loss of signal peptide (p.Met1-A22), glycosylation N-linked (GlcNAc...) asparagine (p.N75) and a partial loss (p.C87-K138) of disulfide bond domain (p.C87-C320). A reduced but high penetrance and variable expressivity of *GRIN2A*-related phenotypes (focal epilepsy with speech disorder and with or without impaired intellectual development) are common and widely described [35].

A *de novo* deletion spanning the exon 3 to exon 5 of the *ZC4H2* gene (NM_018684) was identified in the index case 56-P with global developmental delay, intellectual impairment, and short stature. The *ZC4H2* gene encodes a member of a protein family containing zinc finger domain with four cysteine (C4) and two histidine (H2) residues (OMIM *300897). More than 90% reduction of the mRNA length likely results in its degradation by NMD. Pathogenic structural and sequence variants breaking the *ZC4H2* gene lead to the phenotype of Wieacker-Wolff syndrome both in males and females [36].

## **(C) Novel variants in the candidate genes**

***GRIN3B***

A *de novo* nonsense variant in the *GRIN3B* gene, NM_138690.3:c.931C>T, p.(Gln311Ter), was found in the index case 63-P with the clinical presentation of delayed psychomotor development, abnormal facial appearance and relative macrocephaly. The *GRIN3B* gene encodes a subunit of N-methyl-D-aspartate receptors which form ion channels with the widespread distribution throughout the CNS. Along with GRIN1 and GRIN2A subunits, it regulates membrane permeability and electrical activity of the brain [37; 38]. To date, only *de novo* or inherited missense variants have been proposed as a risk factor especially for neuropsychiatric conditions [39]. *In silico* prediction tools (NMDetective and MutationTaster 2021) predict the incomplete degradation of abnormal transcripts with PTCs (Additional File 5); however, its deleteriousness remains controversial. Previous studies did not support the conclusion for a possible relationship between the rare *GRIN3B* gene variants and intellectual impairment or other NDDs due to its specific expression in motor neurons [38; 40]. Further complementary studies and periodical variant reanalyses may help to elucidate its possible role in the pathogenesis of NDDs. As the *GRIN3B* gene might still be considered as the candidate gene for NDDs, the additional *in silico* analyses using the PANTHER^TM^ Functional classification including the *GRIN3B* gene and its top 10 interaction partners were performed based on the STRING Interaction Network. The *GRIN3B* gene was assigned in all PANTHER^TM^ Ontologies, suggesting its important role in cellular signalling as a transmembrane signal receptor. The overrepresentation analysis on the network including the *GRIN3B* gene and its top 10 predicted interaction partners revealed its significant involvement in 35 biological processes (PANTHER^TM^ GO-Slim Biological Process) with the lowest FDRs in the synaptic transmission and signalling and the regulation of both these processes. The members of the analysed network predominantly function as ion channels and transmembrane signal receptors involved in the neurotransmission (26 GO terms, PANTHER GO-Slim Molecular Function; 1 term, PANTHER Protein Class). They are enriched in the CNS in the postsynaptic densities (31 GO terms, PANTHER GO-Slim Cellular Component resulting from the PANTHER Overrepresentation Test).

***ASAP1***

A novel *de novo* variant in the candidate gene *ASAP1,* NM_018482.4:c.1867C>T, was uncovered in the index case 73-P with the clinical manifestation of predominantly behavioural abnormalities (ASD, absent speech and aggressive behaviour). The encoded protein induces hydrolysis of GDP bound to ARF proteins. It participates in the cytoskeletal and membrane remodelling [41. Using the integrated engine Franklin (Genoox) its pathogenicity is suggested, applying the ACMG criteria PM2, PP2, PP3 and manually added PM6. To date, there is still lacking direct evidence on its functional role and possible clinical consequences. Only a limited number of missense variants are uploaded in the ClinVar database, none of them are reported as P or LP. The adjacent variant NM_018482.4(ASAP1):c.1858G>A has been classified as a VUS (updated on 7^th^ February 2023). The complementary *in silico* analyses using the PANTHER^TM^ Functional classification including the *ASAP1* gene and its top 10 interaction partners were performed based on the STRING Interaction Network. Unlike the *GRIN3B* gene, the *ASAP1* gene was assigned to only three of five PANTHER^TM^ Ontologies (Molecular Function, Pathway). It remains unclassified in the PANTHER^TM^ Ontologies Biological Process, Cellular Component or Protein Class, highlighting the need for further transcriptomic and proteomic analyses. The overrepresentation analysis on the network including the *ASAP1* gene and its top 10 predicted interaction partners uncovered its significant involvement in 19 biological processes (PANTHER^TM^ GO-Slim Biological Process) related to the intracellular protein transport. The members of the analysed network participate in the nucleotide binding (10 GO terms, PANTHER GO-Slim Molecular Function). They are enriched in the cell membrane and periphery as well as in endosomes (6 GO terms; PANTHER GO-Slim Cellular Component) and have significant role in the integrin signalling pathway (4 terms, PANTHER Pathway) based on the outputs of the PANTHER Overrepresentation Test. Recent studies indirectly suggest its possible contribution in the pathogenesis of ASD or other psychiatric disorders through the interaction with known genes which are involved in neuronal development and functioning [ 42; 43]. Further complementary analyses will be required to elucidate its role, if any, in the pathogenesis of NDDs.

# **References**

| 1 | Wayhelova M, Vallova V, Broz P, Mikulasova A, Loubalova D, Filkova H, et al. Novel de novo pathogenic variant in the GNAI1 gene as a cause of severe disorders of intellectual development. J Hum Genet. 2022;67:209-14. |
| --- | --- |
| 2 | Becker LL, Dafsari HS, Schallner J, Abdin D, Seifert M, Petit F, et al. The clinical-phenotype continuum in DYNC1H1-related disorders—genomic profiling and proposal for a novel classification. J Hum Genet. 2020;65:1003-17. |
| 3 | Cappuccio G, Sayou C, Tanno P, Tisserant E, Bruel AL, Kennani S, et al. De novo SMARCA2 variants clustered outside the helicase domain cause a new recognizable syndrome with intellectual disability and blepharophimosis distinct from Nicolaides–Baraitser syndrome. Genet Med. 2020;22:1838-50. |
| 4 | Al-Sayed MD, Al-Zaidan H, Albakheet A, Hakami H, Kenana R, Al-Yafee Y, et al. Mutations in NALCN Cause an Autosomal-Recessive Syndrome with Severe Hypotonia, Speech Impairment, and Cognitive Delay. Am J Hum Genet. 2013;93:721-6. |
| 5 | Gerber S, Alzayady K, Burglen L, Brémond-Gignac D, Marchesin V, Roche O, et al. Recessive and Dominant De Novo ITPR1 Mutations Cause Gillespie Syndrome. Am J Hum Genetics. 2016;98:971-80. |
| 6 | Dudding TE, Friend K, Schofield PW, Lee S, Wilkinson IA, Richards RI. Autosomal dominant congenital non-progressive ataxia overlaps with the SCA15 locus. Neurology. 2004;63:2288-92. |
| 7 | Hamdan FF, Myers CT, Cossette P, Lemay P, Spiegelman D, Laporte AD, et al. High Rate of Recurrent De Novo Mutations in Developmental and Epileptic Encephalopathies. Am J Hum Genet. 2017;101:664-85. |
| 8 | Fan H, Ye X, Shi L, Yin W, Hua B, Song G, et al. Mutations in the EDA gene are responsible for X-linked hypohidrotic ectodermal dysplasia and hypodontia in Chinese kindreds. Eur J Oral Sci. 2008;116:412-7. |
| 9 | Liu Y, Lv Y, Zarrei M, Dong R, Yang X, Higginbotham EJ, et al. Chromosomal microarray analysis of 410 Han Chinese patients with autism spectrum disorder or unexplained intellectual disability and developmental delay. HPJ Genom Med. 2022;7:1. |
| 10 | Fan X, Wang Y, Fan Y, Du H, Luo N, Zhang S, et al. TCOF1 pathogenic variants identified by Whole-exome sequencing in Chinese Treacher Collins syndrome families and hearing rehabilitation effect. Orphanet J Rare Dis. 2019;14:178. |
| 11 | Pilotto S, Speranzini V, Marabelli C, Rusconi F, Toffolo E, Grillo B, et al. LSD1/KDM1A mutations associated to a newly described form of intellectual disability impair demethylase activity and binding to transcription factors. Hum Mol Genet. 2016;25:2578-87. |
| 12 | Wilson HL, Wong ACC, Shaw SR, Tse WY, Stapleton GA, Phelan MC, et al. Molecular characterisation of the 22q13 deletion syndrome supports the role of haploinsufficiency of SHANK3/PROSAP2 in the major neurological symptoms. J Med Genet. 2003;40:575-84. |
| 13 | Betancur C, Buxbaum JD. SHANK3 haploinsufficiency: a “common” but underdiagnosed highly penetrant monogenic cause of autism spectrum disorders. Mol Autism. 2013;4:17. |
| 14 | Rodan LH, Spillmann RC, Kurata HT, Lamothe SM, Maghera J, Jamra RA, et al. Phenotypic expansion of CACNA1C-associated disorders to include isolated neurological manifestations. Genet Med. 2021;23:1922-32. |
| 15 | Schanze I, Bunt J, Lim JWC, Schanze D, Dean RJ, Alders M, et al. NFIB Haploinsufficiency Is Associated with Intellectual Disability and Macrocephaly. Am J Hum Genet. 2018;103:752-68. |
| 16 | Lindeboom RGH, Vermeulen M, Lehner B, Supek F. The impact of nonsense-mediated mRNA decay on genetic disease, gene editing and cancer immunotherapy. Nat Genet. 2019;51:1645-51. |
| 17 | Xu Q, Liu YY, Wang X, Tan GH, Li HP, Hulbert SW, et al. Autism-associated CHD8 deficiency impairs axon development and migration of cortical neurons. Mol Autism. 2018;9:65. |
| 18 | Bernier R, Golzio C, Xiong B, Stessman HA, Coe BP, Penn O, et al. Disruptive CHD8 Mutations Define a Subtype of Autism Early in Development. Cell 2014;158:263-76. |
| 19 | O’Donnell-Luria AH, Pais LS, Faundes V, Wood JC, Sveden A, Luria V, et al. Heterozygous Variants in KMT2E Cause a Spectrum of Neurodevelopmental Disorders and Epilepsy. Am J Hum Genet. 2019;104:1210-22. |
| 20 | Vilboux T, Ciccone C, Blancato JK, Cox GF, Deshpande C, Introne WJ, et al. Molecular Analysis of the Retinoic Acid Induced 1 Gene (RAI1) in Patients with Suspected Smith-Magenis Syndrome without the 17p11.2 Deletion. PLoS One. 2011;6:e22861. |
| 21 | Williams SR, Zies D, Mullegama SV, Grotewiel MS, Elsea SH Smith-Magenis Syndrome Results in Disruption of CLOCK Gene Transcription and Reveals an Integral Role for RAI1 in the Maintenance of Circadian Rhythmicity. Am J Hum Genet. 2012;90:941-9. |
| 22 | Dubruc E, Putoux A, Labalme A, Rougeot C, Sanlaville D, Edery P. A new intellectual disability syndrome caused by CTNNB1 haploinsufficiency. Am J Med Genet. 2014;164A:1571-5. |
| 23 | van Bon BWM, Hoischen A, Hehir-Kwa J, de Brouwer APM, Ruivenkamp C, Gijsbers ACJ, et al. Intragenic deletion in DYRK1A leads to mental retardation and primary microcephaly. Clin Gen. 2011;79:296-9. |
| 24 | Marcogliese PC, Shashi V, Spillmann RC, Stong N, Rosenfeld JA, Koenig MK, et al. IRF2BPL Is Associated with Neurological Phenotypes. Am J Hum Genet. 2018;103:245-60. |
| 25 | Sinha Ray S, Dutta D, Dennys C, Powers S, Roussel F, Lisowski P, et al. Mechanisms of IRF2BPL-related disorders and identification of a potential therapeutic strategy. Cell Rep. 2022;41:111751. |
| 26 | Philip N, Chabrol B, Lossi AM, Cardoso C, Guerrini R, Dobyns WB, et al. Mutations in the oligophrenin-1 gene (OPHN1) cause X linked congenital cerebellar hypoplasia. J Med Genet. 2003;40:441-6. |
| 27 | Guterman EL, Yurgionas B, Nelson AB. Pearls & Oy-sters: Episodic ataxia type 2. Neurology. 2016;86:e239-41. |
| 28 | Heidet L, Morinière V, Henry C, De Tomasi L, Reilly ML, Humbert C, et al. Targeted Exome Sequencing Identifies PBX1 as Involved in Monogenic Congenital Anomalies of the Kidney and Urinary Tract. J Am Soc Nephrol. 2017;28:2901-14. |
| 29 | Slavotinek A, Risolino M, Losa M, Cho MT, Monaghan KG, Schneidman-Duhovny D, et al. De novo, deleterious sequence variants that alter the transcriptional activity of the homeoprotein PBX1 are associated with intellectual disability and pleiotropic developmental defects. Hum Mol Genet. 2017;26:4849-60. |
| 30 | De Rubeis S, He X, Goldberg AP, Poultney CS, Samocha K, Ercument Cicek A, et al. Synaptic, transcriptional and chromatin genes disrupted in autism. Nature. 2014;515:209-15. |
| 31 | Lessel D, Gehbauer C, Bramswig NC, Schluth-Bolard C, Venkataramanappa S, van Gassen KLI, et al. BCL11B mutations in patients affected by a neurodevelopmental disorder with reduced type 2 innate lymphoid cells. Brain. 2018;141:2299-311. |
| 32 | Eto K, Machida O, Yanagishita T, Shimojima Yamamoto K, Chiba K, Aihara Y, et al. Novel BCL11B truncation variant in a patient with developmental delay, distinctive features, and early craniosynostosis. Hum Genome Var. 2022;9:43. |
| 33 | Johansson S, Berland S, Gradek GA, Bongers E, de Leeuw N, Pfundt R, et al. Haploinsufficiency of MEIS2 is associated with orofacial clefting and learning disability. Am J Med Genet A. 2014;164A:1622-6. |
| 34 | Verheije R, Kupchik GS, Isidor B, Kroes HY, Lynch SA, Hawkes L, et al. Heterozygous loss-of-function variants of MEIS2 cause a triad of palatal defects, congenital heart defects, and intellectual disability. Eur J Hum Genet. 2019;27:278-90. |
| 35 | Carvill GL, Regan BM, Yendle SC, O'Roak BJ, Lozovaya N, Bruneau N, et al. GRIN2A mutations cause epilepsy-aphasia spectrum disorders. Nat Genet. 2013;45:1073-6. |
| 36 | Frints SGM, Hennig F, Colombo R, Jacquemont S, Terhal P, Zimmerman HH, et al. Deleterious de novo variants of X‐linked ZC4H2 in females cause a variable phenotype with neurogenic arthrogryposis multiplex congenita. Hum Mutat. 2019;40:2270-85. |
| 37 | Andersson O, Stenqvist A, Attersand A, von Euler G. Nucleotide Sequence, Genomic Organization, and Chromosomal Localization of Genes Encoding the Human NMDA Receptor Subunits NR3A and NR3B. Genomics. 2001;78:178-84. |
| 38 | Niemann S, Landers JE, Churchill MJ, Hosler B, Sapp P, Speed WC, et al. Motoneuron-specific NR3B gene: No association with ALS and evidence for a common null allele. Neurology. 2008;70:666-76. |
| 39 | Hornig T, Grüning B, Kundu K, Houwaart T, Backofen R, Biber K, et al. GRIN3B missense mutation as an inherited risk factor for schizophrenia: whole-exome sequencing in a family with a familiar history of psychotic disorders. Genet Res (Camb). 2017;99:e1. |
| 40 | Tarabeux J, Kebir O, Gauthier J, Hamdan FF, Xiong L, Piton A, et al. Rare mutations in N-methyl-D-aspartate glutamate receptors in autism spectrum disorders and schizophrenia. Transl Psychiatry. 2011;1:e55. |
| 41 | Curtis J, Luo Y, Zenner HL, Cuchet-Lourenço D, Wu C, Lo K, et al. Susceptibility to tuberculosis is associated with variants in the ASAP1 gene encoding a regulator of dendritic cell migration. Nat Genet. 2015;47:523-7. |
| 42 | Irimia M, Weatheritt RJ, Ellis JD, Parikshak NN, Gonatopoulos-Pournatzis T, Babor M, et al. A Highly Conserved Program of Neuronal Microexons Is Misregulated in Autistic Brains. Cell. 2014;159:1511-23. |
| 43 | Lin GN, Song W, Wang W, Wang P, Yu H, Cai W, et al. De novo mutations identified by whole-genome sequencing implicate chromatin modifications in obsessive-compulsive disorder. Sci Adv. 2022;8:eabi6180. |
