# Supplementary material for: Exome sequencing improves the molecular diagnostics of paediatric unexplained neurodevelopmental disorders
Source: Orphanet J Rare Dis. 2024 Feb 6;19:41. doi: 10.1186/s13023-024-03056-6 (PMC10845791; doi:10.1186/s13023-024-03056-6)
Supplement: Supplementary file 7 — Additional file 7: Molecular characterization of variants with borderline classification of pathogenicity (VUS-LP) and their clinical consequences. [file 13023_2024_3056_MOESM7_ESM.docx]

# **Additional File 7: Molecular characterization of variants with borderline classification of pathogenicity (VUS-LP) and their clinical consequences**

The summary of the description using the HGVS nomenclature, *in silico* predictions of the molecular consequences and related phenotypic manifestations are available in Additional File 5. The CNVs mentioned in the index cases 25-P and 47-P suggesting the possible epistatic effects on clinical manifestation are available in Additional File 9b.

***HUWE1***

A novel *de novo* variant altering the X-linked gene *HUWE1* (NM_031407.7:c.11832C>G) was detected in the index case 5-P with global developmental delay, short stature and facial abnormalities. Although it matches the clinical phenotype of *HUWE1-*related disorders, it alters neither a structurally nor functionally important domain. Therefore, further analysis of X-chromosome inactivation or transcriptomic profiling is recommended to specify the clinical relevance of this novel variant.

***KDM3B***

A *de novo* missense variant in the proximal part of the *KDM3B* gene (NM_016604.4:c.370C>A; exon 3) was found in the index case 26-P with intellectual impairment, autism spectrum disorder (ASD) and muscle hypotonia. The *KDM3B* gene encodes a demethylase which removes methyl residues from H3K9, causing transcriptional activation [1]. The phenotypic spectrum of these features has been recently summarized as a rare Diets-Jongmans syndrome; however, all causative variants have been located outside the exon 3 so far [2].

***PIK3R1***

A *de novo* missense variant in the exon 3 of the *PIK3R1* gene (NM_181523.3:c.1693A>G) was identified in the index case 58-P1, who also carried a familial causative *PTCHD1* gene variant (NM_173495.3:c.1765G>T) . The *PIK3R1* gene encodes a regulatory subunit of phosphatidylinositol 3-kinase, which is involved in growth signalling pathways [3]. While somatic variants located in the exon 3 are involved in malignant transformation, the pathogenicity of germline missense variants in this exon remains unresolved [4]. In general, germline causative variants within the *PIK3R1* gene are responsible for the phenotypic manifestation of autosomal dominant disorders such as SHORT syndrome and inborn errors of immunity, as well as autosomal recessive agammaglobulinemia 7 [5; 6; 7].

***CREBBP***

A novel variant in the *CREBBP* gene of paternal origin, NM_004380.3:c.5131A>G, was identified in the index case 30-P. The variant is located within the exon 30, breaking the structurally and functionally important zinc finger domain. Recurrent adjacent variants, c.5128T>C and c.5129G>A, are classified as pathogenic. The phenotypic spectrum related to variants in exons 30 and 31 has been recently defined as a rare Menke-Hennekam syndrome (MHS) type 1, with a clearly distinguishable clinical presentation from Rubinstein-Taybi syndrome type 1 [8]. However, the pathogenicity of the c.5131A>G variant is disputable due to the asymptomatic father 30-M. Moreover, to date, the vast majority of *CREBBP* variants related to MHS were of *de novo* origin. Further studies will be required to elucidate asymptomatic carriers and to identify possible genetic modifiers.

***TAOK1***

A novel variant in the *TAOK1* gene of paternal origin, NM_020791.4:c.593A>C, was uncovered in the index case 47-P with the ASD, intellectual impairment and behavioural abnormalities [9]. The recently reported reduced penetrance and phenotypic variability greatly extends the phenotypic spectrum for *de novo* and inherited rare *TAOK1* variants [10]. As the index case 47-P is also the carrier of low-penetrant, recurrent 22q11.21 microduplication (LCR22A-LCR22D) of maternal origin, the possible epistatic effects on the presentation of phenotypic abnormalities should be considered [11].

***PGK1***

A recurrent X-linked variant of maternal origin affecting the *PGK1* gene (NM_000291.4:cc.392A>G; variant #0000682667 in the Leiden Open Variation Database) was detected in the index case 54-P with neurological abnormalities, which leads to gait disturbances, postural and resting tremor. This variant results in a lysine to arginine substitution, p.(Lys131Arg) with a predicted loss of modified residue N6-acetyllysine or N6-malonyllysine in the β strand (Lys131-Gly132-Lys133). The *PGK1* gene encodes an enzyme with a dual function, serving as both the first ATP-generating glycolytic enzyme and a protein kinase involved in the maintenance of cell homeostasis by regulating cell metabolism and autophagy [12]. The PGK deficiency leads to a highly variable phenotype including haemolytic anaemia, myopathy, with neurological involvement [ 13].

***ZGRF1***

A recurrent haplotype NM_018392.5:c.4087G>A;142C>A of the *ZGRF1* gene of paternal origin was identified in the index case 25-P. It has been suggested as a candidate haplotype for childhood apraxia of speech in a multigenerational family, however, its co-segregation with the observed phenotype was incomplete [14]. Therefore, its own impact on the manifestation of the phenotype is plausible. Moreover, the index case 25-P is a carrier of 6q26 and 16q22.1q22.2 microduplications of maternal origin, which may suggest the epistatic effects of these rare genetic hits on the phenotype of ASD and developmental delay [15].

# **References**

| 1 | Kim J, Kim K, Eom GH, Choe N, Kee HJ, Son H, et al. Kook H, Chakravarti D, Seo S KDM3B Is the H3K9 Demethylase Involved in Transcriptional Activation of lmo2 in Leukemia. Mol Cell Biol. 2012;32:2917-33. |
| --- | --- |
| 2 | Diets IJ, van der Donk R, Baltrunaite K, Waanders E, Reijnders MRF, Dingemans AJM, et al. De Novo and Inherited Pathogenic Variants in KDM3B Cause Intellectual Disability, Short Stature, and Facial Dysmorphism. Am J Hum Genet. 2019;104:758-66. |
| 3 | Vanhaesebroeck B, Welham MJ, Kotani K, Stein R, Warne PH, Zvelebil MJ, et al. p110δ, a novel phosphoinositide 3-kinase in leukocytes. Proc Natl Acad Sci U S A. 1997;94:4330-5. |
| 4 | Liu Y, Wang D, Li Z, Li X, Jin M, Jia N, et al. Pan-cancer analysis on the role of PIK3R1 and PIK3R2 in human tumors. Sci Rep. 2022;12:5924. |
| 5 | Dyment DA, Smith AC, Alcantara D, Schwartzentruber JA, Basel-Vanagaite L, Curry CJ, et al. Mutations in PIK3R1 Cause SHORT Syndrome. Am J Hum Genet. 2013;93:158-66. |
| 6 | Deau MC, Heurtier L, Frange P, Suarez F, Bole-Feysot C, Nitschke P, et al. A human immunodeficiency caused by mutations in the PIK3R1 gene. J Clin Invest. 2014;124:3923-8. |
| 7 | Tang P, Upton JEM, Barton-Forbes MA, Salvadori MI, Clynick MP, Price AK, et al. Autosomal Recessive Agammaglobulinemia Due to a Homozygous Mutation in PIK3R1. J Clin Immunol. 2018;38:88-95. |
| 8 | Menke LA, van Belzen MJ, Alders M, Cristofoli F, Ehmke N, Fergelot P, et al. CREBBP mutations in individuals without Rubinstein-Taybi syndrome phenotype. Am J Med Genet A. 2016;170:2681-93. |
| 9 | Woerden GM, Bos M, Konink C, Distel B, Avagliano Trezza R, Shur NE, et al. TAOK1 is associated with neurodevelopmental disorder and essential for neuronal maturation and cortical development. Hum Mutat. 2021;42:445-59. |
| 10 | Hunter JM, Massingham LJ, Manickam K, Bartholomew D, Williamson RK, Schwab JL, et al. Inherited and de novo variants extend the etiology of TAOK1-associated neurodevelopmental disorder. Cold Spring Harb Mol Case Stud. 2022;8:a006180. |
| 11 | Kylat RI. 22q11.2 Microduplication: An Enigmatic Genetic Disorder. J Pediatr Genet. 2018;7:138-42. |
| 12 | Qian X, Li X, Lu Z. Protein kinase activity of the glycolytic enzyme PGK1 regulates autophagy to promote tumorigenesis. Autophagy. 2017;13:1246-7. |
| 13 | Beutler E. PGK deficiency. Br J Haematol. 2007;136:3-11. |
| 14 | Peter B, Wijsman EM, Nato AQ, Matsushita MM, Chapman KL, Stanaway IB, et al. Genetic Candidate Variants in Two Multigenerational Families with Childhood Apraxia of Speech. PLoS One. 2016;11:e0153864. |
| 15 | Parenti I, Rabaneda LG, Schoen H, Novarino G. Neurodevelopmental Disorders: From Genetics to Functional Pathways. Trends Neurosci. 2020;43:608-21. |
