# Supplementary material for: Exome sequencing improves the molecular diagnostics of paediatric unexplained neurodevelopmental disorders
Source: Orphanet J Rare Dis. 2024 Feb 6;19:41. doi: 10.1186/s13023-024-03056-6 (PMC10845791; doi:10.1186/s13023-024-03056-6)
Supplement: Supplementary file 8 — Additional file 8: Molecular characterization of secondary findings in the “medically-actionable” genes on the ACMG list. [file 13023_2024_3056_MOESM8_ESM.docx]

# **Additional File 8: Molecular characterization of secondary findings in the “medically-actionable” genes on the ACMG list**

***BRCA1* and *BRCA2***

The highest proportion of SF, 71.4% (5/7), was identified in the *BRCA1* and *BRCA2* genes, which are well-known as risk factors for hereditary breast and/or ovarian cancer. Causative variants in the *BRCA1* gene were found in four individuals, two index cases and two parents. A recurrent 4-bp deletion *BRCA1* gene variant, NM_007294.4:c.3700_3704del (rs80357609), was identified in two siblings 43-P1, 43-P2 and their mother 43-M. Another other recurrent *BRCA1* gene splice acceptor variant, NM_007294.4:c.5407-2A>G (rs80358002), was carried by healthy mother 35-M. Finally, a recurrent variant in the *BRCA2* gene, NM_000059.4:c.2808_2811del (rs80359351), was identified in the healthy mother 68-M.

***TGFBR1***

Another subgroup of genes on the ACMG list [1] includes those associated with aortopathies. A pathogenic variant in the *TGFBR1* gene, NM_004612.4:c.1133A>G, was detected in a healthy father 36-F. The variants alters protein-kinase domain (ranging from amino-acid residue Ile205 to amino-acid residue Leu495) of the encoded protein. The ClinVar database categorizes it as a VUS based on one incomplete submission; however, the Franklin engine (Genoox) classified it as likely pathogenic applying the PM2, PP2 and PP3 rules.

***HFE***

A homozygosity for the *HFE* gene variant NM_000410.4:c.845G>A was the reason to report it as the SF in the index case 51-P. The correlation between the *HFE*-related phenotype and the clinical manifestation of the index case 51-P (multiple congenital abnormalities, congenital heart disorder, hypotonia, spasticity and mild intellectual impairment) is disputable and rather unlikely [2]. His parents 51-M and 51-F, as well as his brother 51-S are unaffected carriers of this variant.

# **References**

| 1. | Miller DT, Lee K, Abul-Husn NS, Amendola LM, Brothers K, Chung WK, et al. ACMG SF v3.1 list for reporting of secondary findings in clinical exome and genome sequencing: A policy statement of the American College of Medical Genetics and Genomics (ACMG). Genet Med. 2022;24:1407-14. |
| --- | --- |
| 2. | Pilling LC, Tamosauskaite J, Jones G, Wood AR, Jones L, Kuo C, et al. Common conditions associated with hereditary haemochromatosis genetic variants: cohort study in UK Biobank. BMJ. 2019;364:k5222. |
