# Supplementary material for: Exome sequencing improves the molecular diagnostics of paediatric unexplained neurodevelopmental disorders
Source: Orphanet J Rare Dis. 2024 Feb 6;19:41. doi: 10.1186/s13023-024-03056-6 (PMC10845791; doi:10.1186/s13023-024-03056-6)
Supplement: Supplementary file 10 — Additional file 10: The SNV and CNV detection and prioritization. [file 13023_2024_3056_MOESM10_ESM.docx]

#### **Additional File 10: The SNV and CNV detection and prioritization**

### ***SNV and CNV calling***

Single-nucleotide variants (SNVs) and insertion/deletion variants (indels) were called using the VarScan v2.4.4 (released 25^th^ July 2019; https://github.com/dkoboldt/varscan) with parameters: min-coverage, 20; min-var-freq, 0.1; P-value, 0.5; min-avg-qual, 10. VCF files of index cases and corresponding parents were merged to streamline the variant prioritization and to assess the parental segregation of familial variants. Only SNVs and indels passing the quality filter (a minimal quality of coverage ≥20X, base quality ≥10, mapping quality ≥5) and with an alternative allele frequency (AAF, the fraction of reads with the variant) ≥10% per sample, P-value (Fisher exact test) <0.05 were considered for further variant filtering and prioritization.

Copy-number variations (CNVs) were called using two different bioinformatics pipelines. The first approach was based on the depth calculation and normalization using the R software v3.6.0 (<https://www.r-project.org/>; Rsamtools, Granges packages) in covered exons. Those exons which failed the mappability criteria (lower than 0.75 defined using 35-mer mappability score from UCSC genome browser) were excluded from the analysis. The read depth coverage base line was created using at least six samples (pooled reference). Then the algorithm compared each sample to each. The ratio of expected reads to real number of reads was calculated to estimate a gain or loss in any specific locus defined by target.

The second approach for CNV calling was performed by a custom pipeline CNVRobot v3.3 or higher (https://github.com/AnetaMikulasova/CNVRobot). Briefly, GATK tools (https://github.com/broadinstitute/gatk/) were used for the processing of bam files and data denoising. CNVs and long continuous stretches of homozygosity (LCSH)/losses of heterozygosity (LOH) were called using a custom R-based segmentation and filtered by parameters as follows: CNVs; ≥50 bp and two intervals, ≤-0.5 Log2 Ratio (L2R) for losses and >0.3 L2R for gains. LOH; ≥5 Mb and 10,000 intervals. Unaffected unrelated sex-matching individuals (at least eleven males and eleven females) served as controls for data denoising. The CNV and LCSH/LOH calling using CNVRobot were not performed in the subset of families included in the parental sample pooling design as it initially required index and parental samples for pedigree datasets.

### ***Variant prioritization and classification***

Trio-based approach was proved to be useful for filtering only variants with at least a 20% AAF in index cases. The >20% AAF threshold was also set for parental samples to identify secondary findings (SF) including pathogenic (P) and likely pathogenic (LP) variants in “ACMG” genes [1], with additional P and LP variants in the *CFTR* gene and *F5* variant (NM_000130.5):c.1601G>A, known as Factor V Leiden (FVL). However, this step in the variant filtering was modified for the pooling design strategy. In this approach, the >20% AAF threshold was set only in index cases. The thresholds for SF, P and LP variants in the *CFTR* gene and FVL variant only in parental pools were set at >15% for the AAF in parental two-sample pools and >10% for the AAF in parental three-sample pools.

The Ensembl Variant Effect Predictor v105 [2] was used to prioritize variants with “moderate” or “high” consequences (IMPACT) for further filtering steps. Only rare recurrent variants with allele frequencies less than or equal to 5% or novel variants with unknown allele frequency in the non-Finnish European population (Genome Aggregation Database, gnomAD; https://gnomad.broadinstitute.org/) were considered for the evaluation. Variants were selected using Locus Reference Genomics (LRG) or Canonical Transcripts for reporting clinically relevant sequence variants. Only variants with the “pathogenic” and/or “likely pathogenic” clinical significance based on the current version of the ClinVar database [3] or novel/novel candidate variants in OMIM “morbid” genes were prioritized for the evaluation. The novel variants were considered as candidates only if they were found in a gene involved in the brain or embryonic development, disrupted a structurally or functionally important gene region with a high evolutional conservation. Mitochondrial variants were analysed separately based on the comparison of AAF between index cases and their mothers. After filtering steps, the prioritized variants were visually inspected in the Integrative Genomics Viewer (IGV; v 2.8.6.) [4].

The causative (P or LP) SNVs, indels and intragenic CNVs were analysed and classified using the integrated engines Franklin (Genoox) and VarSome and in silico prediction tools beyond their scope if they were relevant for the nature of variants (NMDetective and NMDesc Predictor for variants which introduced premature termination codon (PTC); Human Splicing Finder and SpliceAI for variants altering canonical splice-donor and splice-acceptor sites) (Additional File 5, Sheet 1-4).

The general information about genes was obtained from the OMIM database [5]. Initially, the variant pathogenicity was assessed using the updated ACMG guidelines. Another set of *in silico* prediction tools served for the evaluation of truncating variants – NMDEsc Predictor [6], NMDetective [7], and splicing variants – Human Splicing Finder [8], SpliceAI [ 9] and CADD using a model GRCh38-v1.6 with the recommended “PHRED-scaled” score ≥20 [10]. Then, the genotype-phenotype correlation was critically evaluated using clinical records and relevant medical and scientific literature. To summarize, only P and LP variants which passed the filtering steps and evaluation criteria were then reported to clinicians for genetic counselling.

CNVs were prioritized according to technical thresholds from two independent bioinformatics algorithms: reads ratios (RR) ≤0.7 for losses and ≥1.3 for gains (Genovesa); log2 ratios (L2R) ≤-0.5 for losses and ≥0.35 for gains (CNVRobot). Only CNVs encompassing OMIM “morbid” genes or candidate genes or classified as P or LP in dbVar database (https://github.com/ncbi/dbvar) were prioritized for further analysis. The presence of LCSH/LOH was evaluated after the manual curation, which compared outputs from the homozygosity mapping from SNV and indel analysis and chromosomal microarray analysis with cut-off of ≥5 Mb. The size of referred CNVs were then specified on the exon level after the manual curation of mapped targeted regions in the IGV.

# **References**

| 1 | Miller DT, Lee K, Abul-Husn NS, Amendola LM, Brothers K, Chung WK, et al. ACMG SF v3.1 list for reporting of secondary findings in clinical exome and genome sequencing: A policy statement of the American College of Medical Genetics and Genomics (ACMG). Genet Med. 2022;24:1407-14. |
| --- | --- |
| 2 | McLaren W, Gil L, Hunt SE, Riat HS, Ritchie GRS, Thormann A, et al. The Ensembl Variant Effect Predictor. Genome Biol. 2016;17:122. |
| 3 | Landrum MJ, Lee JM, Benson M, Brown GR, Chao C, Chitipiralla S, et al. ClinVar: improving access to variant interpretations and supporting evidence. Nucleic Acids Res. 2018;46:D1062-7. |
| 4 | Robinson J, Thorvaldsdóttir H, Winckler W, Guttman M, Lander ES, Getz G, et al. Integrative genomics viewer. Nat Biotechnol. 2011;29:24-6. |
| 5 | Hamosh A, Scott AF, Amberger JS, Bocchini CA, McKusick VA. Online Mendelian Inheritance in Man (OMIM), a knowledgebase of human genes and genetic disorders. Nucleic Acids Res. 2005;33:D514-7. |
| 6 | Coban-Akdemir Z, White JJ, Song X, Jhangiani SN, Fatih JM, Gambin T, et al. Identifying Genes Whose Mutant Transcripts Cause Dominant Disease Traits by Potential Gain-of-Function Alleles. Am J Hum Genet. 2018;103:171-87. |
| 7 | Lindeboom RGH, Vermeulen M, Lehner B, Supek F. The impact of nonsense-mediated mRNA decay on genetic disease, gene editing and cancer immunotherapy. Nat Genet. 2019;51:1645-51. |
| 8 | Desmet FO, Hamroun D, Lalande M, Collod-Béroud G, Claustres M, Béroud C. Human Splicing Finder: an online bioinformatics tool to predict splicing signals. Nucleic Acids Res. 2009;37:e67. |
| 9 | Jaganathan K, Kyriazopoulou Panagiotopoulou S, McRae JF, Darbandi SF, Knowles D, Li YI, et al. Predicting Splicing from Primary Sequence with Deep Learning. Cell. 2019;176:535-548.e24. |
| 10 | Rentzsch P, Witten D, Cooper G, Shendure J, Kircher M. CADD: predicting the deleteriousness of variants throughout the human genome. Nucleic Acids Res. 2019;47:D886-94. |
